# What is the evidence for virtual wards or hospital-at-home care pathways for exacerbations of chronic obstructive pulmonary disease? A systematic review and meta-analysis

**DOI:** 10.1136/bmjresp-2025-003611

**Published:** 2026-04-10

**Authors:** Bushra Alenazi, Christopher Hatton, Elizabeth Sapey

**Affiliations:** 1 Respiratory Care—College of Applied Medical Sciences, Imam Abdulrahman Bin Faisal University, Jubail, Saudi Arabia; 2 NIHR Midlands Patient Safety Research Collaboration, Department of Inflammation & Ageing, College of Medicine and Health, University of Birmingham College of Medical and Dental Sciences, Birmingham, UK; 3 Institute of Inflammation and Ageing, University of Birmingham College of Medical and Dental Sciences, Birmingham, UK

**Keywords:** COPD Exacerbations

## Abstract

**Objectives:**

Given increasing interest in admission avoidance, we evaluated the evidence to support virtual wards (VW) and hospital at home (HaH) models of care during exacerbations of chronic obstructive pulmonary disease (ECOPD).

**Design:**

A systematic review and meta-analysis. A comprehensive search of MEDLINE (1946 to March 2024), Embase (1974 to March 2024) and CENTRAL (searched 22 March 2024) was conducted. Risk of bias and a random effects meta-analysis were performed.

**Population:**

Adults with an ECOPD presenting to the hospital or who require hospital-led care.

**Interventions:**

VW: defined as assessments and interventions delivered remotely or HaH (defined as assessments and interventions delivered by healthcare professionals in patient’s homes) care pathways, compared with hospital admission.

**Primary and secondary objectives:**

Safety (mortality rate of all causes, in-patient, 7 days and 30 days) and readmission rate in 7 and 30 days. Length of stay in hospital and changes in pulmonary function tests.

**Results:**

One study assessed VWs (reported in two publications) and 10 assessed HaH. There were no changes in survival or short-term readmission rates attributable to the interventions and no evidence that VW or HaH care pathways reduced the total time a patient spent under hospital-led care, whether at home or in the hospital.

**Conclusions:**

More evidence is needed to support the widespread roll-out of HaH and especially VW pathways for ECOPD.

**PROSPERO registration number:**

https://www.crd.york.ac.uk/PROSPERO/view/CRD42024517565.

**WHAT IS ALREADY KNOWN ON THIS TOPIC**

- Virtual wards and hospital-at-home models are increasingly implemented to manage exacerbations of chronic obstructive pulmonary disease (ECOPD); however, the existing evidence is heterogeneous, and the benefits and effects of these services remain uncertain.

**WHAT THIS STUDY ADDS**

- This systematic review and meta-analysis synthesises existing evidence on virtual wards and hospital-at-home pathways for ECOPD, revealing considerable variation in service models, patient selection criteria and reported outcomes.

**HOW THIS STUDY MIGHT AFFECT RESEARCH, PRACTICE OR POLICY**

- The findings highlight the need for clearer definitions of virtual ward and hospital-at-home services as well as more consistent and standardised outcome reporting. Future research should prioritise outlining the core components of each model, establishing patient selection criteria and evaluating both cost-effectiveness and long-term outcomes.

## Introduction

 Chronic obstructive pulmonary disease (COPD) is a heterogeneous lung condition characterised by chronic breathlessness, progressive airflow obstruction and acute exacerbations requiring additional treatment.^1^ Exacerbations contribute significantly to mortality and declining lung function^2 3^ and are the second leading cause of emergency admissions in the United Kingdom (UK).^4^ These admissions are costly, accounting for 70% of COPD-related expenses and are linked to poor outcomes, including infections, falls, delirium and death.^3 5 6^

Moreover, the global health system is currently under pressure due to an ageing population, rising costs and an increasing demand for healthcare services. Traditional models for providing healthcare are struggling to keep pace with these escalating costs and environmental impacts.^7^ This situation was further exacerbated during the COVID-19 pandemic, which highlighted the urgent need for innovative healthcare solutions and resilient approaches to healthcare delivery.^8^

To address this, alternative service delivery models providing inpatient-level care outside of hospitals have been developed. These models are commonly referred to as virtual wards (VW) or hospital-at-home (HaH) services. They have been used in various forms for several years and may help to prevent unnecessary hospital admissions or enable early discharges. Recently, countries such as the United Kingdom (UK) have seen increased investment and expansion in the provision of home-based inpatient care models.^9 10^

The terms VW and HaH have been used interchangeably and there is debate surrounding the terminology and defining characteristics of these services. A rapid evidence synthesis published in 2023^11^ describes HaH as face-to-face multidisciplinary care delivered in the community as an alternative to inpatient admission, while VW provides acute care at home using technology-enabled remote monitoring and treatment. VW models may combine remote and in-person care, depending on patient needs, and commonly use digital tools such as apps, wearables and oximeters.^11^ Moreover, in the British Geriatric Society (BGS) position statement ‘Right Time, Right Place: Urgent Community-Based Care for Older People’ (2021) outlines that HaH services provide in-person, face-to-face acute care within a patient’s home. These services are delivered by multidisciplinary teams that are equipped to assess, diagnose and treat patients in their home environment. While VW primarily rely on technology and remote monitoring, using digital tools and wearable devices, complemented by multidisciplinary oversight and optional in-home visits.^12^

In the National Health Service (NHS) England’s 2024 Operational Guidance adopts the World Hospital at Home Congress Consensus definition (2023),^13^ describing the VWs as technology-enabled services delivering hospital-level diagnostics, interventions and monitoring. The guidance also states that local services can decide whether to name services VW or HaH services. Moreover, the Hospital at Home Society defines HaH as delivering hospital-level diagnostics (eg, ultrasound) and treatments (eg, IV therapy, oxygen) via multidisciplinary teams who visit the patient in their own home including the provision of 24-hour cover with the ability to respond to urgent visits.^13^ The BGS, the UK Hospital at Home Society, and the Royal College of Physicians London released a joint statement supporting the use of the term ‘Hospital at Home’, rather than ‘VW’ to describe the provision of any hospital-level care at home.^14^

A spectrum of services exist, ranging from remote care to intensive in-person care at home. Both systems require hospital-led coordination, patient review and clear escalation plans. However, depending on the service configuration, they are likely to have substantially different staffing requirements and costs associated. All of these factors are important when planning to adopt a care model into clinical practice. For the purposes of this systematic review (SR), we have opted to refer to services as VWs if the service is primarily enabled by technology and as HaH if it provides face-to-face care. These services have been developed to reduce healthcare costs while improving patient experience,^15^ recognising that patients often prefer to receive care in their homes instead of being hospitalised.^16 17^

Both models can function as early supported discharge (ESD) or admission avoidance (AA) services. ESD facilitates early discharge following initial hospital care, while AA enables direct community admission, bypassing hospitalisation.^18^ Despite their growing adoption,^11 19^ concerns remain regarding their safety, effectiveness, acceptability and cost-efficiency.^11 20 21^

Several SRs have been conducted to assess HaH and VWs in ECOPD, but these reviews were either conducted some time ago, missing more recent studies or focused on one outcome (such as readmissions).^22 23^ A recent SR combined different interventions in ECOPD, including prolonged telemedicine care as an outpatient, when assessing outcomes. This approach makes it difficult to assess whether the currently proposed VW and HaH models of care have evidence to support their use.^24 25^

To address these limitations, this SR differentiates between VW and HaH care pathways and assesses a broad range of clinical parameters as outcomes.

## Methodology

### Protocol and registration

This SR followed the Preferred Reporting Items for Systematic Reviews and Meta-Analyses (PRISMA) 2020 guidelines.^26^ The protocol was registered in PROSPERO (https://www.crd.york.ac.uk/PROSPERO/view/CRD42024517565). Following registration, the protocol was amended to refine the review outcomes; the updated version was approved and is available in PROSPERO.

### Eligibility criteria

Studies were selected according to the PICOS (Population, Intervention, Comparator, Outcomes and Study design) criteria; all criteria and outcomes of interest are presented in table 1.

**Table 1:** Inclusion and exclusion criteria for title, abstract and full-text selection (PICOS)

PICOS	Inclusion criteria	Exclusion criteria
Population	Adults aged 18 years or older with an ECOPD presenting to the hospital or who require hospital-led care.	Paediatric patients and individuals younger than 18 years. Not an ECOPD population or patients who do *not* require hospital-led care.
Intervention	VW or HaH care pathways, including ESD and AA, which provide hospital-led care to patients in their own homes.	Other home or community care systems not used for the treatment of ECOPD. Services that provide long-term telemedicine or remote monitoring for *stable* COPD in outpatient settings. End-of-life care services.
Comparator	Patients who are admitted to the hospital for an ECOPD and who receive usual care as an in-patient.	No comparator group. Studies not comparing outcomes against in-hospital admissions.
Outcomes	Coprimary outcomes: Safety (mortality rate of all causes, in-patient, 7 days and 30 days) Readmission rate in 7 and 30 days. Secondary outcomes: Length of stay in the hospital and length of stay on the VW or HaH care pathway. Exacerbation rates up to 12 months after the index exacerbation. Patient selection criteria for VW Treatments included as part of the care model 5. Changes in physiology, including oxygen saturations and respiratory function	Studies reporting outcomes outside the scope of the predefined primary or secondary outcomes.
Study design	All randomised and non-randomised controlled trials and observational studies with both an intervention and comparator arm were included. There were no restrictions on study dates or languages.	All types of narrative reviews. Case studies and case series (no comparator) Studies with<10 participants (to ensure minimum methodological quality)

PICOS framework: population, intervention (HaH/VW), comparator, outcomes and study design.

AA, admission avoidance; ECOPD, exacerbations of chronic obstructive pulmonary disease; ESD, early supported discharge; HaH, hospital at home; VW, virtual wards.

### Information sources and search strategy

MEDLINE, Embase and CINAHL were selected for their comprehensive coverage of biomedical and community care literature. Additional grey literature and manual searches were conducted covering policy documents, NHS publications, national and international guidelines, and the reference lists of all key studies and reviews were screened to minimise the risk of missing eligible studies. MEDLINE (Ovid, 1946 to March 2024), EMBASE (Ovid, 1974 to March 2024) and the Cochrane Central Register of Controlled Trials (CENTRAL, searched 22 March 2024). The search-combined free-text keywords and Medical Subject Headings (MeSH) terms related to COPD (eg, ‘COPD’, ‘Chronic Obstructive Pulmonary Disease’, MeSH: ‘Pulmonary Disease, Chronic Obstructive’) and virtual models of care (eg, ‘virtual ward’, ‘hospital at home’, ‘telemedicine’, MeSH: ‘Telemedicine’, ‘Home Care Services, Hospital-Based’). Retrieved articles were imported to Rayyan, a web application that facilitates screening for SR.^27^

### Selection process

Abstracts were independently screened by reviewers BA and CH, with any discrepancies resolved by a third reviewer, ES, through consensus. Articles that were marked as ‘included’ or ‘maybe’ underwent a full-text review based on PICOS criteria (see table 1). The outcomes selected were aimed to evaluate the safety and effectiveness of HaH and VW services. While a 30-day cut-off was planned for primary outcomes, most studies reported longer follow-ups; therefore, all relevant data were extracted for subgroup analysis.

### Data collection process

A data extraction table, approved by all reviewers, was used by BA and CH. Both extracted and cross-checked study data, which were synthesised through meta-analysis and narrative review. References were managed using Mendeley.^28^

### Intervention classification

Interventions where either all care was planned virtually or only a baseline assessment was planned to be delivered as face-to-face were deemed to be VW studies. Interventions where healthcare professionals delivered care in the patient’s home were classified as HaH studies. Studies where patients were admitted to the HaH or VW service from the hospital or following an emergency department attendance were classified as ESD. Studies where patients were placed on these services from the community were classified as AA. As there was only one VW study (published over two articles) for the meta-analysis, VW and HaH studies were pooled into a single intervention, but results are also discussed separately.

### Risk of bias

Risk of bias (ROB) was assessed using the Cochrane Risk of Bias Tool V.2 (RoB2) for randomised controlled studies (RCTs) and the Newcastle-Ottawa Scale (NOS) for cohort studies. ROB2 was conducted at the outcome level, specifically for the two co-primary outcomes included in the meta-analysis (mortality and readmission). BA and CH independently assessed each study, with disagreements resolved by a third reviewer (ES).

### Data analysis

Coprimary outcomes (mortality rate and readmission rate) were analysed using meta-analysis. Readmissions were defined as a return to hospital following discharge from VW/HaH or hospital. Patients who returned to hospital prior to discharge from VW/HaH were considered as treatment failure or escalation. RevMan software (V.5.4.1) was used to conduct a meta-analysis of the mortality rates and readmission rates extracted from the included studies. For the meta-analysed outcomes (mortality and readmission), the number of participants included reflected the total number of patients analysed by the authors for that outcome. Where studies reported withdrawals or loss to follow-up, these patients were retained in the denominator in keeping with an intention-to-treat principle. A random effects model was used to account for heterogeneity, and pooled effect sizes were calculated using the Mantel-Haenszel method and are presented with 95% CIs and p values.

Secondary outcomes (length of stay (LoS), respiratory function (forced expiratory volume in 1 second (FEV1), forced vital capacity (FVC), peak expiratory flow rate (PEFR)), oxygen saturation) were synthesised narratively due to substantial clinical and methodological heterogeneity between studies. Descriptive statistics were extracted and reported as presented by the original study authors, and no statistical pooling was performed for these outcomes.

Subgroup patterns were analysed qualitatively by presenting outcomes based on follow-up duration (eg, 1 month, 3-month and 6-month mortality) and by model type (HaH vs VW). Meta-analysis was conducted for mortality and readmission outcomes when at least two studies reported data at the same follow-up point. Due to the limited number of studies for each outcome and the considerable methodological variability, conducting a formal subgroup meta-analysis by model type was not feasible. All data were extracted as reported by the study authors, with no imputation or statistical reconstruction of missing values performed.

### Heterogeneity and reporting publication bias

Statistical heterogeneity among studies was assessed using the I^²^ statistic, τ² and Cochran’s Q test, with heterogeneity interpreted in conjunction with forest plot inspection. τ² was used to estimate between-study variance, with values of zero, indicating no detectable variance across studies. Cochran’s Q test was used to assess the presence of statistical heterogeneity. I^²^ values <40% indicated low heterogeneity, 40%–60% moderate, 60%–90% substantial and >75% high. Funnel plots were also used to assess potential publication bias or small-study effects.

No formal sensitivity analyses were performed due to the limited number of studies contributing to each outcome. The potential removal of individual studies or exclusions based on RoB would likely result in analyses that are unstable or lack informative value. Therefore, heterogeneity was assessed narratively within the Results and Discussion sections.

## Results

### Study selection

**Figure 1:**
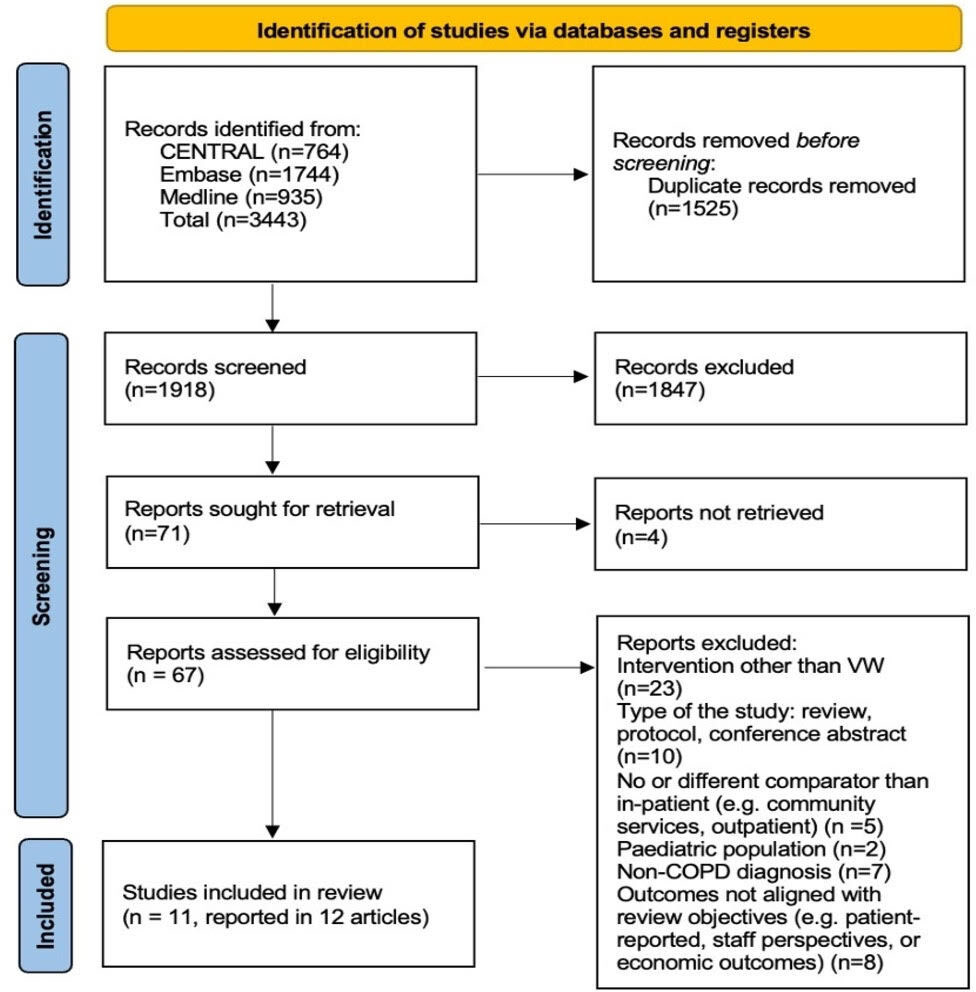
PRISMA flow diagram. This diagram outlining the study selection process for this systematic review. COPD, chronic obstructive pulmonary disease; PRISMA, Preferred Reporting Items for Systematic Reviews and Meta-Analyses; VW, virtual wards.

The search yielded 3443 citations with 1918 screened after removing duplicates. Sixty-seven studies underwent full-text review and 11 studies met the inclusion criteria (reported in 12 articles): 10 RCTs and one observational cohort study. Figure 1 presents the PRISMA flow diagram. Notably, the only study of VW was published in two articles (Jakobsen *et al*^29^ and Schou *et al*^30^) each reported different outcomes.

### Study characteristics

Main study characteristics are summarised in table 2. Additional study-level and aggregated participant information is provided in online supplemental table 1, while primary outcomes are detailed in online supplemental table 2. All studies were based in Europe between 2000 and 2018, mostly UK,^2531^,^35^ two from Denmark (reported in three publications^29 30 36^), and one each from Italy,^37^ Spain^38^ and The Netherlands.^39^ UK studies were single centre, while others were multicentre.

**Table 2:** Summary of the study characteristics of all included studies in this systematic review

Study	Study design	Centre	Geographical location	Income level	Language of publication	Intervention	Patients in each arm	
							I	UC
Cotton *et al*^25^	RCT	Single	Glasgow, UK	High-income	English	HaH ESD	41	40
Skwarska *et al*^31^	RCT	Single	Edinburgh, UK	High-income	English	HaH ESD	122	62
Davies *et al*^32^	RCT	Single	Liverpool, UK	High-income	English	HaH ESD	100	50
Ojoo *et al*^33^	RCT	Single	Hull, UK	High-income	English	HaH ESD	30	30
Díaz Lobato *et al*^38^	RCT	Multi	Madrid, Spain	High-income	English	HaH ESD	20	20
Nissen and Jensen^36^	RCT	Multi	Denmark	High-income	English	HaH ESD	22	22
Ricauda *et al*^37^	RCT	Single	Torino, Italy	High-income	English	HaH ESD	52	52
Ansari *et al*^34^	OCT	Single	Sunderland, UK	High-income	English	HaH AA	30	60
Utens *et al*^39^	RCT	Multi	The Netherlands	High-income	Danish	HaH ESD	70	69
Schou *et al*^30^*	RCT	Multi	Denmark	High-income	English	VW ESD	22	22
Jakobsen *et al*^29^*	RCT	Multi	Denmark	High-income	English	VW ESD	29	28
Echevarria *et al*^35^	RCT	Multi	Newcastle, UK	High-income	English	HaH ESD	60	58

* The same study published with different outcomes reported across two publications.

AA, admission avoidance; ESD, early supported discharge; HaH, hospital at home; I, intervention; OCT, observational cohort study with no randomisation; RCT, randomised controlled study; UC, usual care; VW, virtual wards.

Although 12 papers were included, they represented 11 distinct studies, as the Jakobsen VW study was reported across two publications.^29 30^ Ten studies assessed HaH intervention involving regular home visits, typically daily. A study by Jakobsen *et al*^29^ (published over two papers by Schou *et al*^30^) comprised the only VW study, with a single face-to-face baseline assessment followed by virtual monitoring. All studies were based on ESD, except Ansari *et al*^34^ assessed AA.

Eleven studies were unblinded RCTs,^2529^,^33 35^ and one was an observational cohort study.^34^ Eleven studies were published in English; one Danish^36^ and was translated using Google Translate.^40 41^

In total, 1111 participants (aged 60–80) were included: 628 patients received the intervention (599 HaH, 29 VW).

Most studies used similar criteria to define ECOPD, indicating comparable populations, though eligibility criteria varied (see online supplemental table 1). Timing of intervention differed: some studies did not report this, while others specified initiation within 24 hours (Schou *et al*,^30^ and ‘within 72 hours’ for Cotton *et al*,^25^ or after a minimum of three inpatient days (Utens *et al*^39^)).

Descriptions of interventions were limited. HaH visit frequency was rarely reported, and although some studies (eg, Echevarria *et al*)^35^ mentioned potential multidisciplinary input, but its implementation was unclear.

Our coprimary safety outcomes included all-cause mortality and readmission during HaH/VW compared with usual care at 7 and 30 days, aligned with standard definitions.^35^ However, most reported outcomes at later time points (eg, 2, 3 and 6 months). Therefore, all relevant data were extracted for a subgroup meta-analysis. See online supplemental table 2.

Reasons for treatment failures (escalation from VW to hospital admission before discharge from VW) and readmission (patients in the VW and UC groups readmitted to the hospital after being discharge from the service) were either unreported or non-comparable across groups. For example, in Diaz Lobato *et al*,^38^ therapeutic failure included the need for admission to intensive care and nosocomial infections in the conventional hospitalisation group, while in the HaH group, therapeutic failure was defined as requiring transfer to the hospital.

The LoS was reported in seven studies, but it was often unclear whether this referred to initial hospital admission or time spent in HaH/VW care.

Respiratory function outcomes were reported in several studies, but the measures and findings varied. All outcomes of each study are described in online supplemental table 1.

### Risk of bias

The assessment of the ten RCTs indicated that most trials had either some concerns or a high RoB. The NOS assessment for the observational study showed a high overall high RoB due to lack of comparability and poor follow-up reporting. Full details are provided in online supplemental file 1.

### Coprimary outcomes

Mortality and readmission rates were reported in all studies included in this SR, with the exceptions of Schou *et al*,^30^ which did not report either mortality or readmission rates, and Ansari *et al*,^34^ which reported the mortality rate for both groups but only addressed readmissions in the discussion section for one group only. As a result, Ansari *et al*’s study^34^ was excluded from the meta-analysis on readmissions. It is important to note that the timing of included events varied across the studies.

### Mortality rates

Two studies (HAH: Diaz *et al*^38^ and VW: Jakobsen *et al*^29^) reported 1-month mortality, matching our coprimary outcome. Overall, one patient died in the usual care group in Diaz *et al*^38^ and no patients died in the intervention group. Given these studies included 20 and 29 patients in the intervention arm, respectively, studies were underpowered to report against this outcome.

No differences were found in mortality rates comparing the intervention to usual care for any other timepoints. Three studies reported mortality within 2 months (All HaH: Cotton *et al*,^25^ Skwarska *et al*,^31^ Nissen *et al*^36^) with a pooled relative risk (RR) of 0.41 (95% CIs 0.15 to 1.11), p=0.08) with no observed statistical heterogeneity (I^²^=0%, τ^2^=0.00, χ^2^=1.90, df=2, p=0.39). Six studies reported 3-month mortality (Davis *et al*,^32^ Ojoo *et al*,^33^ Ansari *et al*,^34^ Utens *et al*,^39^ Jakobsen *et al*,^29^ Echevarria *et al*^35^). The pooled RR for these studies was 0.89 (95% CI 0.37 to 2.18), p=0.81, with no observed statistical heterogeneity (I^²^=0%, τ^2^=0.00, χ^2^=0.94, df=3, p=0.82). Two studies reported 6-month mortality (Ricauda *et al*,^37^ Jakobsen *et al*^29^) with a pooled RRs of 0.74 (95% CI 0.38 to 1.47), p=0.39 and no observed statistical heterogeneity (I²=0%, τ^2^=0.00, χ^2^=0.00, df=1, p=0.97).

All timepoints were then combined and overall mortality was assessed with a RR of 0.67 (95% CI 0.42 to 1.08), p=0.10 and no observed statistical heterogeneity (I²=0%, τ^2^=0.00, χ^2^=4.46, df=9, p=0.88). Notably, in all time points I^2^=0%, indicating no observed statistical heterogeneity. However, some variation was noted in the study results. The wide and overlapping CIs seen may reflect the small sample size of included studies.

**Figure 2:**
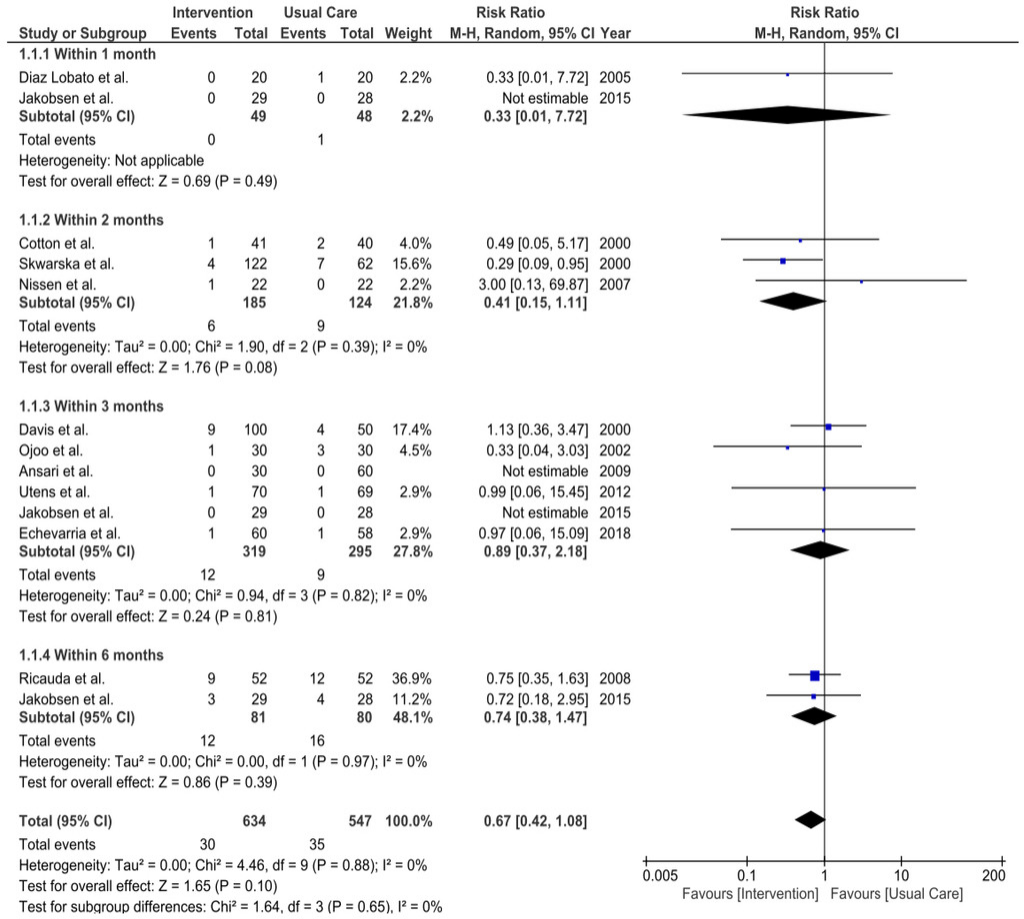
Comparison of the mortality rates between VW versus in-patient care. df, degrees of freedom; I², I-squared statistic; M-H, Mantel-Haenszel; τ^2^, between-study variance estimate, VW, virtual wards.

For a forest plot of these results, see figure 2.

### Readmission rate

All studies except Schou *et al*^30^ and Ansari *et al*^34^ reported readmission rates. Ansari *et al*^34^ addressed readmissions only for one group in the discussion, without details for the hospital group. As a result, this study was excluded from the readmissions meta-analysis. The remaining studies provided readmission rates at different time points. To maintain consistency and include relevant data, we considered all-cause readmissions, regardless of the reason, since some studies reported respiratory and non-respiratory readmissions separately while others grouped them together. None of the individual studies reported a difference in readmission rates between the intervention and usual care group.

One-month readmissions were reported in two studies (HaH: Diaz *et al*^38^ and VW: Jakobsen *et al*^29^), showing no differences, RR 1.38 (95% CI 0.57 to 3.34), p=0.48, with no observed statistical heterogeneity (I²=0%, τ^2^=0.00, χ^2^=0.26, df=1, p=0.61).

Three HaH studies (Cotton *et al*,^25^ Skwarska *et al*,^31^ Nissen *et al*^36^) reported 2-month readmissions, pooled RR of 0.71 (CI 0.49 to 1.03), p=0.07, with no statistically observed heterogeneity (I^²^=0%, τ^2^=0.00, χ^2^=1.41, df=2, p=0.49).

Five studies reported 3-month readmission rates (HaH: Davis *et al*,^32^ Ojoo *et al*,^33^ Utens *et al*,^39^ Echevarria *et al*.^35^ VW: Jakobsen *et al*^29^. The pooled RR was 0.95 (95% CI 0.74 to 1.21), p=0.67, with no observed statistical heterogeneity (I^²^ = 0%, τ^2^=0.00, χ^2^=0.85, df=4, p=0.93).

Two studies reported 6-month readmission (HaH: Ricauda *et al*,^37^ VW: Jakobsen *et al*^29^). Pooled RR was 0.65 (95% CI 0.37 to 1.16), p=0.14, with substantial heterogeneity observed (I^²^=63%, τ^2^ = 0.11, χ^2^=2.67, df=1, p=0.10), although the Cochran’s Q test was non-significant, which likely reflects limited statistical power resulting from the small number of included studies.

**Figure 3:**
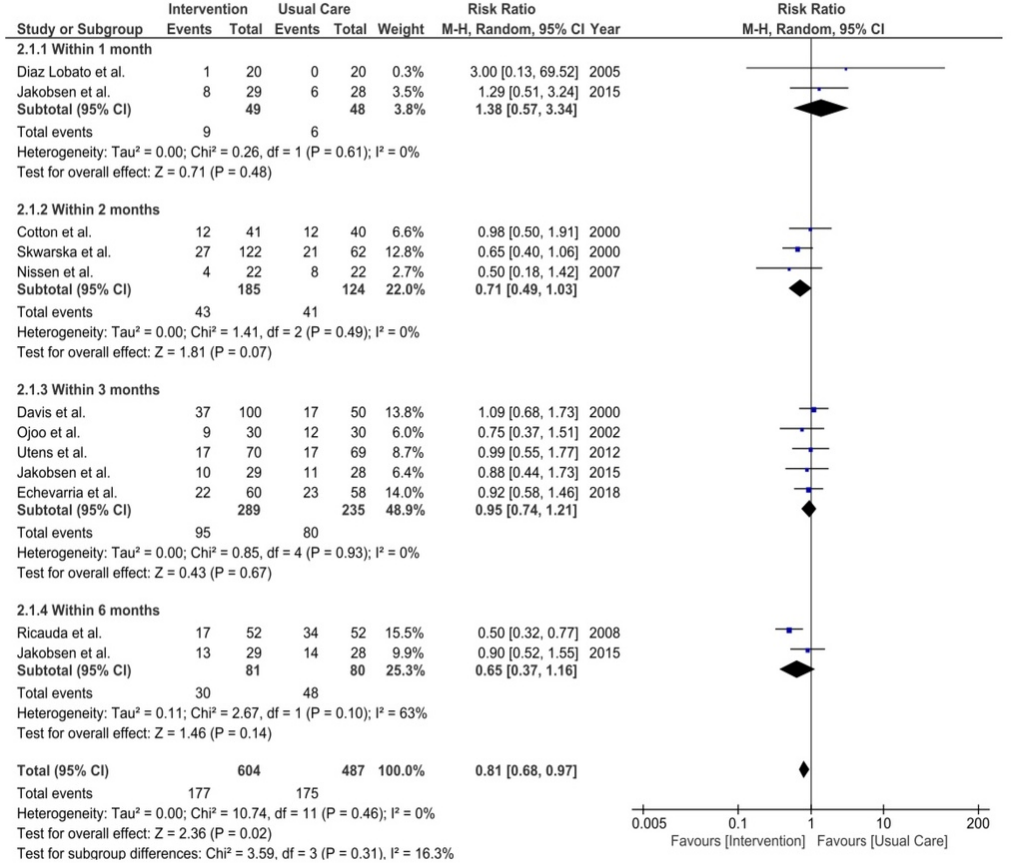
Comparison of the readmission rates between VW versus in-patient care. df, degrees of freedom; I², I-squared statistic; M-H, Mantel-Haenszel; τ^2^, between-study variance estimate, VW, virtual wards.

Pooled analysis across all timepoints showed reduced readmission risk. The RR was 0.81 (95% CI 0.68 to 0.97), p=0.02, with no observed statistical heterogeneity (I^²^=0%, τ^2^=0.00, χ^2^=10.74, df=11, p=0.46). Shorter follow-up periods are more likely to reflect the direct impact of the intervention. Thus, it is improbable that this association is causally linked to the intervention, particularly as the strength of the association was not noticeably higher with shorter follow-up periods. Observed variability and overlapping CIs likely reflect small sample sizes rather than true effect differences. See figure 3 for the forest plot.

### Secondary outcomes

#### Length of stay

LoS was reported in six studies. For HaH services, Cotton *et al*,^25^ Nissen *et al*^36^ and Echevarria *et al*^35^ assessed initial hospital stay only, all showing shorter LoS in the intervention group (eg, Cotton: 3.2 vs 6.1 days; Nissen: 1.3 vs 3.7 days; Echevarria: 1.2 vs 4.1 days).

Skwarska *et al*,^31^ Diaz *et al*,^38^ Echevarria *et al*^35^ and Nissen *et al*^36^ compared LoS for the usual care group and the HaH, including both the initial hospitalisation and time on the HaH/V service. Results varied: Diaz *et al*^38^ reported a shorter LoS for the intervention group, with a mean LoS of 9.2 days (4 days in the hospital and 5 days at home) for the HaH group compared with 12.2 days in the in-patient group. Echevarria *et al*^35^ reported no difference between HaH and usual care groups. Nissen *et al*^36^ and Skwarska *et al*^31^ reported a longer total LoS in the HaH group compared with the usual care group. Nissen *et al* reported a mean stay of 6.4 days in the HaH group compared with 3.7 days in the usual care group and Skwarska *et al*^31^ reported a total LoS in the HaH group, with a median of 7 days compared with 5 days in the usual care group.

The only VW study (Jakobsen *et al*^29^ reported LoS as the proportion of patients hospitalised >5 days, with lower rates in the intervention group (17.2%) compared with usual care (28.6%)). However, duration on VW and detailed LoS data were not reported. No studies reported exacerbation rates up to 12 months after the index exacerbation, or the treatments included as part of the care model.

#### Changes in respiratory function

The variability of lung function measures in exacerbations of COPD limited the ability of the included studies to reliably detect or interpret physiological changes.^42^ FEV₁ was reported in six studies; however, one was a substudy (Schou *et al*^30^) derived from a larger trial (Jakobsen *et al*^29^) and included a smaller patient population. Although both studies are described separately due to differences in sample size and analysis, they are not treated as fully independent sources. For HaH studies, Ansari *et al*^34^ reported significant improvement in both the HaH and in-patient groups. Davis *et al*^32^ and Ojoo *et al*^33^ reported no difference in HaH or usual care group compared with baseline. Skwarska *et al*^31^ and Schou *et al*^30^ reported an improvement in FEV1 in the intervention group only—HaH and VW, respectively—although no significant difference was reported between the groups. For the VW study, Jakobsen *et al*^29^ reported no FEV1 differences.

FVC was reported in Ojoo *et al*^33^ (HaH) and Jakobsen *et al*,^29^ and both reported no difference in change in FVC. PEFR was only reported by Skwarska *et al*^31^ with improvement in both groups. Oxygen saturation was reported in three studies. Jakobsen *et al*^29^ found no significant difference. Schou *et al*^30^ observed an improvement in the VW group only. Skwarska *et al*^31^ noted a slight increase in the in-patient group only. Overall, no study reported a significant difference between groups.

#### Publication bias

Funnel plots were used to assess the likelihood of publication bias for mortality and readmission outcomes. For mortality, the funnel plot demonstrated a degree of asymmetry, suggesting a possibility of publication bias, favouring studies with positive effects. The funnel plot for readmission rates showed symmetry, indicating a low likelihood of publication bias. Funnel plots are provided in online supplemental figures 1 and 2.

## Discussion

### Summary of key findings

This SR aimed to evaluate the effectiveness and safety of VW and HaH services for ECOPD. We initially planned to assess VW and HaH separately due to their significant differences in required staff, costs and the level of continuous monitoring provided by VW. Although both models share the common goal of delivering acute hospital-led care in the patient’s home, there are substantial conceptual and operational differences between them that justify a separate evaluation. However, only one study identified in this SR used predominantly remote care, and therefore all studies are grouped together in quantitative synthesis. The one study exploring the impact of predominantly remote care reported shorter initial hospital stays but found no differences in the predefined outcomes, including 30-day readmission/treatment failure, mortality, need for ventilation (manual, mechanical or non-invasive ventilation (NIV)), health-related quality of life or user satisfaction. Overall, evidence supporting remote care models, including optimal selection criteria and duration, remains limited.

Most studies included provided more intensive face-to-face care at home, which we refer to as HaH. These studies tended to be small, and most were of low or moderate quality. Variability existed in timing, selection and services. No significant difference in 30-day mortality or readmission was observed. HaH was linked to shorter initial admissions, though total care duration was not consistently reduced.

Eleven out of 12 assessed the impact of ESD rather than AA. Therefore, it is unclear whether AA HaH models of care are safe or effective.

Meta-analysis of the coprimary 30-day outcomes showed no significant differences in survival or readmission. The pooled meta-analysis for all timepoints showed no difference in mortality between control and intervention groups, although event rates were low. A reduction in readmissions was observed when combining all time periods, largely driven by studies assessing 3–6 month outcomes. Given that acute interventions primarily affect short-term outcomes, readmission reductions at 6 months are less likely attributable to the intervention.

### Relationship with the existing literature

There was no evidence that VW or HaH reduced overall time under hospital-led care (either at home or in hospital). Four previous SRs examined using HaH or VW models of care for patients with ECOPD^2243^,^45^ but did not distinguish between high-intensity HaH and more remote VW models—an important distinction for service planning. For example, an SR published in 2024 included telemonitoring for up to 1-year postadmission, and 3 monthly telephone calls for up to 1 year^24^ rather than acute ESD or AA strategies for ECOPD. Furthermore, previous SRs did not specifically define patient outcomes and this impacts on how studies are interpreted. For example, when Echevarria *et al*^46^ defined readmission as a return to the hospital after discharge from the VW, excluding hospital admissions, which occurred during the HaH intervention (which they classified as an escalation of care), HaH was associated with significant decrease in readmissions. However, when all hospital returns, including those from HaH before being discharged, were considered readmissions, no difference in readmission rates was observed between the usual care and intervention groups. Our current review includes some new studies not covered in previous SRs.

We have excluded two studies that were included in previous SRs. A study by Hernandez *et al*^46^ was excluded as patients were eligible for inclusion if they attended Accident and Emergency (A&E) with ECOPD that did not require admission to hospital. We also exclude a study by Nicholson *et al*^47^ as it included patients referred from the outpatient department and therefore did not meet our criteria for selection.

### Implications for clinicians and policymakers

Clinical teams and policymakers should be aware that, at the current time, there is limited evidence to support VW or HaH care models and the studies reported to date focus more on ESD rather than AA. Reassuringly, the evidence that exists does not suggest patient harm from VW or HaH models, including no difference in mortality or short-term readmission rates.

Proponents of VW and HaH care models would hope that their use could reduce the strain across healthcare systems and lower hospital bed occupancy. However, the results of studies included in this review were variable. HaH and VWs may reduce initial hospital stay prior to HaH or VW admission, but their impact on total length of stay, including time spent on HaH or VWs, is more uncertain.

This review did not identify a clear benefit of VW or HaH models of care for people attending hospital with ECOPD, and further research is warranted. Notably, most studies did not provide clear and explicit admission criteria, and future research could seek to understand which patients may benefit the most from these services. In addition to the quantitative outcomes reported in this review, it is also important to consider cost-effectiveness and qualitative outcomes such as patient experience.

Therefore, at the current time, healthcare staff, organisations, funders and policymakers developing or commissioning VW or HaH services should be aware that they are introducing services without a clear evidence base to support them. There are no equity analyses to determine whether any groups might be disadvantaged by such a service or health economic analyses to determine cost implications for the service. As with all new services, clinical vigilance is needed with careful service evaluations conducted to review patient flow, outcomes and costs. Setting standardised inclusion and exclusion criteria for each disease, along with ongoing evaluation after implementation, is essential to ensure patient safety, clinical effectiveness and alignment with health system goals.

### Strengths and limitations

Regarding the strengths of this SR, it followed the PRISMA 2020 guidelines. Moreover, unlike previous SRs, we clearly defined VW and HaH care pathways, allowing a more precise assessment of the evidence supporting each model. However, the studies reported to date have been small, often single centre and heterogeneous in both design and outcomes. Most studies evaluated services that provided substantial face-to-face care in the patient’s home, with only one study reporting results from predominantly remote care models. These factors, collectively, represent important limitations of this SR. In addition, the Danish paper was translated using machine-translation tools without review by a native speaker. This presents a potential limitation, as relying solely on machine translation may introduce inaccuracies in data extraction.

### Future research recommendations

Current studies supporting the use of both VW and HaH are limited in number, small in size, mostly outdated and vary greatly in their inclusion criteria, outcomes measured and application of interventions. This makes it difficult to generalise the findings, meaning that the existing evidence is insufficient to warrant the widespread implementation of these services. Future research should aim to clearly define the core components of each model, identify appropriate patient selection criteria and evaluate cost-effectiveness and long-term benefits. High-quality, multicentre trials are essential to determine which model offers the greatest value at both the clinical and system levels.

## Conclusion

There is limited high-quality evidence exploring the impact of VW and HaH services on mortality and readmission rates. This SR highlights the need for high-quality evidence to understand the effectiveness, selection criteria, limitations and capacity required. Many healthcare systems worldwide have started expanding their VW and HaH services, yet questions remain surrounding their effectiveness and safety.

## Supplementary material

10.1136/bmjresp-2025-003611 online supplemental file 1

10.1136/bmjresp-2025-003611 online supplemental file 2

## Data Availability

Data sharing is not applicable as no datasets generated and/or analysed for this study.
