# Supplementary material for: What is the evidence for virtual wards or hospital-at-home care pathways for exacerbations of chronic obstructive pulmonary disease? A systematic review and meta-analysis
Source: BMJ Open Respir Res. 2026 Apr 10;13(1):e003611. doi: 10.1136/bmjresp-2025-003611 (PMC13084816; doi:10.1136/bmjresp-2025-003611)
Supplement: online supplemental file 1 [file bmjresp-13-1-s001.docx]

**Supplementary File 1 – Methods Appendix**

1. **Literature search strategies - Search Terms**

The searches included combining free text terms around the three search components ‘Chronic obstructive pulmonary disease’, ‘virtual ward’, ‘hospital at home’, and ‘Telehealth’. These were then adapted to be used in other databases. For instance, the search strategy included: (Tele*health OR Telemonitor* OR Telemedicine OR Telecare OR Teleconsultation OR Remote* healthcare OR Digital* health OR e-medicine OR virtual* ward*s OR Virtual care OR hospital at home* OR HAH OR Hospital @ home) AND (Chronic obstructive pulmonary disease OR COPD OR Emphysema OR Chronic airway obstruction OR Chronic bronch*itis).

1. **Embase Search Terms**

Embase <1974 to 2024 March 20>

| #Search | Search Term | Results |
| --- | --- | --- |
| 1 | Tele*health.mp. [mp=title, abstract, heading word, drug trade name, original title, device manufacturer, drug manufacturer, device trade name, keyword heading word, floating subheading word, candidate term word] | 34905 |
| 2 | Telemonitor*.mp. [mp=title, abstract, heading word, drug trade name, original title, device manufacturer, drug manufacturer, device trade name, keyword heading word, floating subheading word, candidate term word] | 8505 |
| 3 | Telemedicine.mp. [mp=title, abstract, heading word, drug trade name, original title, device manufacturer, drug manufacturer, device trade name, keyword heading word, floating subheading word, candidate term word] | 63324 |
| 4 | Telecare.mp. [mp=title, abstract, heading word, drug trade name, original title, device manufacturer, drug manufacturer, device trade name, keyword heading word, floating subheading word, candidate term word] | 3062 |
| 5 | Teleconsultation.mp. [mp=title, abstract, heading word, drug trade name, original title, device manufacturer, drug manufacturer, device trade name, keyword heading word, floating subheading word, candidate term word] | 17940 |
| 6 | Remote* healthcare.mp. [mp=title, abstract, heading word, drug trade name, original title, device manufacturer, drug manufacturer, device trade name, keyword heading word, floating subheading word, candidate term word] | 467 |
| 7 | Digital* health.mp. [mp=title, abstract, heading word, drug trade name, original title, device manufacturer, drug manufacturer, device trade name, keyword heading word, floating subheading word, candidate term word] | 13824 |
| 8 | e-medicine.mp. [mp=title, abstract, heading word, drug trade name, original title, device manufacturer, drug manufacturer, device trade name, keyword heading word, floating subheading word, candidate term word] | 126 |
| 9 | virtual* ward*s.mp. [mp=title, abstract, heading word, drug trade name, original title, device manufacturer, drug manufacturer, device trade name, keyword heading word, floating subheading word, candidate term word] | 135 |
| 10 | Virtual care.mp. [mp=title, abstract, heading word, drug trade name, original title, device manufacturer, drug manufacturer, device trade name, keyword heading word, floating subheading word, candidate term word] | 2175 |
| 11 | hospital at home*.mp. [mp=title, abstract, heading word, drug trade name, original title, device manufacturer, drug manufacturer, device trade name, keyword heading word, floating subheading word, candidate term word] | 1237 |
| 12 | HAH.mp. [mp=title, abstract, heading word, drug trade name, original title, device manufacturer, drug manufacturer, device trade name, keyword heading word, floating subheading word, candidate term word] | 701 |
| 13 | Hospital @ home.mp. [mp=title, abstract, heading word, drug trade name, original title, device manufacturer, drug manufacturer, device trade name, keyword heading word, floating subheading word, candidate term word] | 464 |
| 14 | Chronic obstructive pulmonary disease.mp. [mp=title, abstract, heading word, drug trade name, original title, device manufacturer, drug manufacturer, device trade name, keyword heading word, floating subheading word, candidate term word] | 100806 |
| 15 | COPD.mp. [mp=title, abstract, heading word, drug trade name, original title, device manufacturer, drug manufacturer, device trade name, keyword heading word, floating subheading word, candidate term word] | 125362 |
| 16 | Emphysema.mp. [mp=title, abstract, heading word, drug trade name, original title, device manufacturer, drug manufacturer, device trade name, keyword heading word, floating subheading word, candidate term word] | 56827 |
| 17 | Chronic airway obstruction.mp. [mp=title, abstract, heading word, drug trade name, original title, device manufacturer, drug manufacturer, device trade name, keyword heading word, floating subheading word, candidate term word] | 508 |
| 18 | Chronic bronch*itis.mp. [mp=title, abstract, heading word, drug trade name, original title, device manufacturer, drug manufacturer, device trade name, keyword heading word, floating subheading word, candidate term word] | 19849 |
| 19 | 1 or 2 or 3 or 4 or 5 or 6 or 7 or 8 or 9 or 10 or 11 or 12 or 13 | 118038 |
| 20 | 14 or 15 or 16 or 17 or 18 | 214305 |
| 21 | 19 and 20 | 1744 |

1. **Ovid MEDLINE Search Terms**

Ovid MEDLINE(R) <1946 to March Week 3 2024>

| #Search | Search Term | Results |
| --- | --- | --- |
| 1 | Tele*health.mp. [mp=title, abstract, heading word, drug trade name, original title, device manufacturer, drug manufacturer, device trade name, keyword heading word, floating subheading word, candidate term word] | 18503 |
| 2 | Telemonitor*.mp. [mp=title, abstract, heading word, drug trade name, original title, device manufacturer, drug manufacturer, device trade name, keyword heading word, floating subheading word, candidate term word] | 2960 |
| 3 | Telemedicine.mp. [mp=title, abstract, heading word, drug trade name, original title, device manufacturer, drug manufacturer, device trade name, keyword heading word, floating subheading word, candidate term word] | 58645 |
| 4 | Telecare.mp. [mp=title, abstract, heading word, drug trade name, original title, device manufacturer, drug manufacturer, device trade name, keyword heading word, floating subheading word, candidate term word] | 1075 |
| 5 | Teleconsultation.mp. [mp=title, abstract, heading word, drug trade name, original title, device manufacturer, drug manufacturer, device trade name, keyword heading word, floating subheading word, candidate term word] | 1959 |
| 6 | Remote* healthcare.mp. [mp=title, abstract, heading word, drug trade name, original title, device manufacturer, drug manufacturer, device trade name, keyword heading word, floating subheading word, candidate term word] | 411 |
| 7 | Digital* health.mp. [mp=title, abstract, heading word, drug trade name, original title, device manufacturer, drug manufacturer, device trade name, keyword heading word, floating subheading word, candidate term word] | 13400 |
| 8 | e-medicine.mp. [mp=title, abstract, heading word, drug trade name, original title, device manufacturer, drug manufacturer, device trade name, keyword heading word, floating subheading word, candidate term word] | 78 |
| 9 | virtual* ward*s.mp. [mp=title, abstract, heading word, drug trade name, original title, device manufacturer, drug manufacturer, device trade name, keyword heading word, floating subheading word, candidate term word] | 76 |
| 10 | Virtual care.mp. [mp=title, abstract, heading word, drug trade name, original title, device manufacturer, drug manufacturer, device trade name, keyword heading word, floating subheading word, candidate term word] | 1843 |
| 11 | hospital at home*.mp. [mp=title, abstract, heading word, drug trade name, original title, device manufacturer, drug manufacturer, device trade name, keyword heading word, floating subheading word, candidate term word] | 868 |
| 12 | HAH.mp. [mp=title, abstract, heading word, drug trade name, original title, device manufacturer, drug manufacturer, device trade name, keyword heading word, floating subheading word, candidate term word] | 604 |
| 13 | Hospital @ home.mp. [mp=title, abstract, heading word, drug trade name, original title, device manufacturer, drug manufacturer, device trade name, keyword heading word, floating subheading word, candidate term word] | 304 |
| 14 | Chronic obstructive pulmonary disease.mp. [mp=title, abstract, heading word, drug trade name, original title, device manufacturer, drug manufacturer, device trade name, keyword heading word, floating subheading word, candidate term word] | 68987 |
| 15 | COPD.mp. [mp=title, abstract, heading word, drug trade name, original title, device manufacturer, drug manufacturer, device trade name, keyword heading word, floating subheading word, candidate term word] | 66103 |
| 16 | Emphysema.mp. [mp=title, abstract, heading word, drug trade name, original title, device manufacturer, drug manufacturer, device trade name, keyword heading word, floating subheading word, candidate term word] | 39701 |
| 17 | Chronic airway obstruction.mp. [mp=title, abstract, heading word, drug trade name, original title, device manufacturer, drug manufacturer, device trade name, keyword heading word, floating subheading word, candidate term word] | 377 |
| 18 | Chronic bronch*itis.mp. [mp=title, abstract, heading word, drug trade name, original title, device manufacturer, drug manufacturer, device trade name, keyword heading word, floating subheading word, candidate term word] | 10772 |
| 19 | 1 or 2 or 3 or 4 or 5 or 6 or 7 or 8 or 9 or 10 or 11 or 12 or 13 | 79870 |
| 20 | 14 or 15 or 16 or 17 or 18 | 130657 |
| 21 | 19 and 20 | 935 |

1. **CENTRAL Search Terms**

Last Saved: 22/03/2024

| #Search | Search Term | Results |
| --- | --- | --- |
| 1 | MeSH descriptor: [Telemedicine] explode all trees | 5249 |
| 2 | MeSH descriptor: [Remote Consultation] explode all trees | 480 |
| 3 | MeSH descriptor: [Digital Health] explode all trees | 43 |
| 4 | MeSH descriptor: [Home Care Services, Hospital-Based] explode all trees | 278 |
| 5 | MeSH descriptor: [Pulmonary Disease, Chronic Obstructive] explode all trees | 7996 |
| 6 | MeSH descriptor: [Bronchitis, Chronic] explode all trees | 207 |
| 7 | MeSH descriptor: [Emphysema] explode all trees | 317 |
| 8 | "virtual ward":ti,ab,kw OR "hospital at home":ti,ab,kw OR "remote monitoring":ti,ab,kw OR "home hospitalization":ti,ab,kw | 1440 |
| 9 | ((home OR digital OR virtual OR tele*) NEAR/4 (care OR service* OR ward* OR team*)):ti,ab,kw | 16438 |
| 10 | ((remote OR digital OR electronic) NEAR/4 (follow-up OR monitoring)):ti,ab,kw | 3900 |
| 11 | (COPD or "chronic obstructive pulmonary disease" or "chronic bronchitis" or emphysema):ti,ab,kw | 25640 |
| 12 | #1 OR #2 OR #3 OR #4 OR #8 OR #9 OR #10 | 23771 |
| 13 | #5 OR #6 OR #7 OR #11 | 26190 |
| 14 | #12 AND #13 | 764 |

1. **Risk of Bias assessment**:

The risk of bias results is provided for each study including the tool that was used.

1. **The Cochrane Risk of Bias 2 tool** **(ROB 2) –** for the following Randomized Controlled Trials (RCTs)
2. **Mortality Rate:**

| 1. Cotton et al., 2000 | | |
| --- | --- | --- |
| Risk of Bias (ROB2) | | |
| Bias Domain | **Consensus**  **judgement** | **Supporting information** |
| 1. Bias due to randomization Process | Low Risk | Randomisation was carried out independently of the clinical team using a pre-specified random treatment allocation schedule.  Baseline characteristics were similar between groups, suggesting successful randomisation. These points also apply to the mortality outcome, which is objective and unlikely to be influenced by any minor baseline imbalance. |
| 1. Bias due to deviations from intended intervention | Some Concerns | Blinding not possible due to nature of intervention.  One patient was randomised to usual care but later opted for early supported discharge - it is likely this patient opted for this option as they were aware of the trial investigation.  This deviation only occurred in one patient and analysis was ITT, and mortality is an objective outcome that is unlikely to be affected by this deviation |
| 1. Bias due to missing outcome data | Some Concern | Five patients withdrew from the early discharge group, and one patient withdrew from the conventional care group.  The study does not explicitly address whether missing data could have biased results. Although mortality is an objective measure and could likely have been obtained, the imbalance in withdrawals leaves “Some concerns” about the risk of bias. |
| 1. Bias due to measurement of the outcome | Low Risk | Limited information provided about how outcomes were measured/ data were collected, but mortality is an objective outcome and not influenced by assessor knowledge of intervention group. |
| 1. Bias due to selection of the reported result | Low Risk | No pre-specified statistical plan, but all outcomes were objective and unadjusted, reducing selective reporting risk. Mortality was reported transparently, and there is no indication that multiple outcomes were measured but selectively reported. |
| Overall risk of bias | Some Concerns | |
| The key issues relate to withdrawals and lack of blinding, but given that mortality is an objective outcome, these are unlikely to substantially bias the results. Therefore, the overall judgement remains “Some concerns.” | | |

| 2. Skwarska et al., 2000 | | |  |
| --- | --- | --- | --- |
| Risk of Bias (ROB2) | | |  |
| Bias Domain | **Consensus judgement** | **Supporting information** |  |
| 1. Bias due to randomization Process | Some Concerns | The study states that randomization was done using computer-generated numbers. However, it does not clearly state whether allocation was concealed, which raises concerns. The imbalance in baseline characteristics (for example, more severe breathlessness in the admitted group) could suggest an issue with randomization, although it may also be due to chance. Since mortality is an objective outcome, this imbalance could still influence results if the more severe patients were systematically allocated to one arm. Therefore, “Some concerns” applies to the mortality outcome. |  |
| 1. Bias due to deviations from intended intervention | Low Risk | It is impossible to blind participants due to the nature of the intervention. However, there is no evidence that deviations occurred in a way that would systematically affect outcomes. The study analysed results on an intention-to-treat basis, which reduces the risk that deviations influenced the mortality outcome. |  |
| 1. Bias due to missing outcome data | High Risk | Six patients in the hospital group were lost to follow-up, while none were missing in the early discharge group. This substantial imbalance raises the possibility of bias, particularly because mortality was a rare event. If any of the lost patients had died, this would directly affect the outcome and bias the comparison. Therefore, the risk of bias for mortality is high. |  |
| 1. Bias due to measurement of the outcome | Low Risk | The study does not provide detailed information about how outcomes were measured, but mortality is an objective endpoint and unlikely to be influenced by assessor awareness of group allocation. There is no indication that mortality would be assessed differently between groups. Therefore, risk of bias is low for this domain. |  |
| 1. Bias due to selection of the reported result | Low Risk | No pre-specified analysis plan was mentioned, but the reported outcomes are standard for COPD trials and mainly objective, including mortality. There is no evidence of selective reporting or that multiple mortality measures were collected but not reported. This domain is therefore low risk for the mortality outcome. |  |
| Overall risk of bias | High Risk | |  |
| The primary concerns relate to the substantial imbalance in missing outcome data (6 vs. 0), which could plausibly bias mortality estimates. Although the outcome itself is objective, missing mortality data in one arm makes the overall risk of bias high. | | |  |

| 3. Davies et al., 2000 | | |
| --- | --- | --- |
| Risk of Bias (ROB2) | | |
| Bias Domain | **Consensus judgement** | **Supporting information** |
| 1. Bias due to randomization Process | Low Risk | Patients were randomized in a 2:1 ratio using blinded sealed envelopes, and baseline characteristics were comparable. |
| 1. Bias due to deviations from intended intervention | Low Risk | Due to the nature of the intervention, blinding was not feasible. No clear deviations from intervention because of trial context. Some patients were appropriately admitted to hospital following randomisation to hospital at home; this is likely what would have occurred in a real-life setting. |
| 1. Bias due to missing outcome data | Some Concerns | 5% loss to follow-up in the home care group vs. 10% in the hospital group could introduce bias if missing data were related to mortality or worsening health.  It is possible that patients lost to follow-up may have died, meaning missingness could depend on the true value of the outcome. Although mortality is likely to have been obtained from objective sources, this is not explicitly stated. Therefore, “Some concerns” applies to the mortality outcome. |
| 1. Bias due to measurement of the outcome | Low Risk | The authors do not clearly describe how outcomes were measured. However, mortality is an objective event, and it is unlikely that it would be ascertained differently between groups. Most outcomes were objective, and lack of blinding is unlikely to have influenced mortality assessment. Therefore, the risk of bias for this domain is low. |
| 1. Bias due to selection of the reported result | Low Risk | No pre-specified analysis plan was reported, but mortality was presented as a straightforward and unadjusted comparison, with no indication of multiple testing or selective reporting. There is no evidence that mortality was measured but not reported. Thus, this domain is low risk for the mortality outcome. |
| 1. Overall risk of bias | Some Concerns | |
| The main issue is the imbalance in missing data between study arms (10% vs 5%). Although mortality is objective and likely to have been collected accurately, missingness may still introduce some uncertainty. Therefore, the overall judgement for mortality is “Some concerns.” | | |

| 4. Ojoo et al., 2002 | | |
| --- | --- | --- |
| Risk of Bias (ROB2) | | |
| Bias Domain | **Consensus judgement** | **Supporting information** |
| 1. Bias due to randomization Process | Low Risk | Patients were randomized using sealed envelopes. No significant differences in baseline characteristics were reported. |
| 1. Bias due to deviations from intended intervention | Low Risk | Blinding was not possible due to the nature of the intervention. However, there were no evident deviations from the intended treatment as a result of the trial.  Patients who were readmitted and unable to complete the trial had their results excluded. Since the number of withdrawals was the same in both study arms, this is unlikely to have significantly affected the overall findings. |
| 1. Bias due to missing outcome data | Some Concerns | The study reports that 27 patients in each arm completed the trial, but it is unclear whether any outcome data was missing for them. While there is no strong evidence of bias due to missing data, withdrawals were equal across groups, reducing potential impact. However, missing data might be linked to outcomes, as patients who were readmitted could have had a higher risk of mortality. Despite this, the small sample size and balanced withdrawals make it unlikely that missing data significantly influenced the study’s conclusions. |
| 1. Bias due to measurement of the outcome | Low Risk | There is limited detail about how outcomes were measured, but mortality is an objective event unlikely to differ by knowledge of intervention. There is no indication that outcome assessors would influence mortality data. |
| 1. Bias due to selection of the reported result | Low Risk | No pre-specified analysis plan was mentioned, but objective outcomes were reported without evidence of selective reporting. Mortality appears fully reported. |
| Overall risk of bias | Some Concerns | |
| Unclear missing data handling introduces some uncertainty, but balanced withdrawals and the objective nature of mortality reduce the overall risk. | | |

| 5. Díaz Lobato et al., 2005 | | |
| --- | --- | --- |
| Risk of Bias (ROB2) | | |
| Bias Domain | **Consensus judgement** | **Supporting information** |
| 1. Bias due to randomization Process | Some Concerns | The study states that randomization occurred but does not describe the method. However, baseline characteristics between groups appear similar, reducing risk. |
| 1. Bias due to deviations from intended intervention | Low Risk | Blinding was not possible. No evidence of deviations from the intended intervention due to trial context. Only one patient in the home group was readmitted. Mortality is objective and unlikely to be influenced. Low risk. |
| 1. Bias due to missing outcome data | Some Concerns | The paper does not provide detailed information on missing data. Mortality was reported as similar between groups, reducing concern, but missing data handling is unclear. |
| 1. Bias due to measurement of the outcome | Low Risk | There is insufficient reporting about how outcomes were collected. Mortality is an objective event unlikely to differ by knowledge of intervention. |
| 1. Bias due to selection of the reported result | Low Risk | No pre-specified analysis plan was mentioned, but objective outcomes were reported without evidence of selective reporting. Mortality appears fully reported. |
| Overall risk of bias | Some Conserns | |
| The poor reporting of measurement methods and unclear handling of missing data elevate the risk of bias for the mortality outcome. | | |

| 1. Nissen et al., 2007 | | |
| --- | --- | --- |
| Risk of Bias (ROB2 - Cluster) | | |
| Bias Domain | **Consensus judgement** | **Supporting information** |
| 1. Bias due to randomization Process | Low Risk | Randomisation was performed blinded by the project nurse. Baseline characteristics were balanced​. |
| 1. Bias due to deviations from intended intervention | Low Risk | Blinding was not possible. Very low readmission numbers and ITT-style reporting indicate no major deviations likely to impact mortality. |
| 1. Bias due to missing outcome data | Low Risk | Missing outcome data were not explicitly reported, but all randomized patients appear in the tables. Mortality is objective, further supporting low risk. |
| 1. Bias due to measurement of the outcome | Low Risk | Mortality is objective. Outcome assessors may have known allocation, but mortality is not susceptible to assessor influence. |
| 1. Bias due to selection of the reported result | Low Risk | No evidence of selective reporting. The outcomes appear fully reported. Low risk. |
| Overall risk of bias | Low Risk | |
| The identified concerns are minor and unlikely to have materially affected the results. | | |

| 1. Ricauda et al., 2008 | | |
| --- | --- | --- |
| Risk of Bias (ROB2) | | |
| Bias Domain | **Consensus judgement** | **Supporting information** |
| 1. Bias due to randomization Process | Low Risk | The study states that randomization was conducted using computer-generated numbers, and allocation was concealed (sealed envelopes). Similar baseline patient characteristics |
| 1. Bias due to deviations from intended intervention | Low Risk | Not possible to blind due to nature of intervention.  No evidence of deviations from intended intervention because of trial context.  Authors state that results analysed on intention to treat basis. Since mortality is objective, deviations unlikely to bias results. |
| 1. Bias due to missing outcome data | Low Risk | Three patients were lost to follow-up, with similar numbers in both groups. Unlikely to influence mortality comparisons. Low risk. |
| 1. Bias due to measurement of the outcome | Low Risk | Mortality measurement was part of a clear 6-month follow-up. Unclear how mortality was obtained, but outcome is objective and unlikely to differ between groups. |
| 1. Bias due to selection of the reported result | Low Risk | The authors state that sample size calculations were used but there is no pre-specified analysis plan provided. There is no evidence that multiple outcome measurements/ times were used.  There is no evidence that multiple analyses were performed. |
| Overall risk of bias | Low Risk | |
| Minor concerns only, with no meaningful impact on mortality. | | |

| 1. Utens et al, 2012 | | |
| --- | --- | --- |
| Risk of Bias (ROB2 - Cluster) | | |
| Bias Domain | **Consensus judgement** | **Supporting information** |
| 1. Bias due to randomization Process | Low Risk | The paper clearly states: Randomisation sequence generated by computer with allocation in sealed envelopes.  Comparable baseline characteristics |
| 1. Bias due to deviations from intended intervention | Low Risk | Blinding not possible due to nature of intervention.  No clear evidence of deviation from intended intervention because of trial context  Authors report that ITT was used. |
| 1. Bias due to missing outcome data | High Risk | High dropout overall (16%), with imbalance: 25% usual care vs 10% intervention. Since mortality could occur among dropouts, this imbalance may bias results. |
| 1. Bias due to measurement of the outcome | Low Risk | Outcome measurement was standardized, using the same procedures for both groups. Mortality unlikely to be influenced. Low risk. |
| 1. Bias due to selection of the reported result | Low Risk | Pre-specified analysis plan in trial protocol.  Pre-specified outcomes  Pre-specified analysis. |
| 1. Overall risk of bias | High Risk | |
| Overall high degree of bias due to the number of patients that withdrew and were lost to follow up. The proportions were different between the intervention and control group suggesting bias. | | |

| 1. Jakobsen, et al, 2015 | | |
| --- | --- | --- |
| Risk of Bias (ROB2) | | |
| Bias Domain | **Consensus judgement** | **Supporting information** |
| 1. Bias due to randomization Process | Low Risk | Randomization was external, with sealed opaque envelopes and concealed allocation. |
| 1. Bias due to deviations from intended intervention | Low Risk | Not possible to blind due to nature of intervention.  Intention-to-treat analysis was conducted​. |
| 1. Bias due to missing outcome data | Low Risk | Missing data were substantial for respiratory function, but mortality data came from registry sources, meaning they were complete and not influenced by dropouts. Therefore, missing data do not bias the mortality outcome. Low risk. |
| 1. Bias due to measurement of the outcome | Low Risk | Mortality measured using registry data, which is objective and consistent. Blinding at assessment is irrelevant. |
| 1. Bias due to selection of the reported result | Low Risk | Published trial protocol available  No evidence multiple outcome measurements used  No evidence multiple analyses performed. |
| Overall risk of bias | Low Risk | |
| Although the study had issues in other outcomes, mortality is unaffected due to objective registry-based ascertainment. | | |

| 1. Echevarria et al., 2018 | | |
| --- | --- | --- |
| Risk of Bias (ROB2) | | |
| Bias Domain | **Consensus judgement** | **Supporting information** |
| 1. Bias due to randomization Process | Low Risk | External independent randomisation with minimisation, researchers blind to allocation. |
| 1. Bias due to deviations from intended intervention | Low Risk | Not possible to blind due to nature of intervention.  Intention to treat analysis used. |
| 1. Bias due to missing outcome data | Low Risk | Some missing cost data, but mortality was fully captured using clear procedures for patients who withdrew. |
| 1. Bias due to measurement of the outcome | Low Risk | Mortality measurement methods were clearly described and consistent. Outcomes obtained from reliable sources. |
| 1. Bias due to selection of the reported result | Low Risk | Pre-specified outcomes/ analysis and trial registered.  No evidence of multiple outcome measures.  No evidence of multiple analyses of the data. |
| Overall risk of bias | Low Risk | |
| No concerns across domains. | | |

**2- Readmission rate:**

| 1. Cotton et al., 2000 | | |
| --- | --- | --- |
| Risk of Bias (ROB2) | | |
| Bias Domain | **Consensus**  **judgement** | **Supporting information** |
| 1. Bias due to randomization Process | Low Risk | Randomisation was carried out independently of the clinical team using a pre-specified random treatment allocation schedule.  Baseline characteristics were similar between groups, suggesting successful randomisation. Any differences observed likely due to chance. |
| 1. Bias due to deviations from intended intervention | Some Concerns | Blinding not possible due to nature of intervention.  One patient was randomised to usual care but later opted for early supported discharge - it is likely this patient opted for this option as they were aware of the trial investigation.  This deviation only occurred in one patient and analysis was ITT.  Comparison of readmissions made using all patients recruited to their initial groups. |
| 1. Bias due to missing outcome data | Some Concern | Five patients withdrew from the early discharge group, and one patient withdrew from the conventional care group.  The study does not explicitly address whether missing data could have biased results.  ITT analysis was performed, reducing the risk of bias. |
| 1. Bias due to measurement of the outcome | Low Risk | Limited information provided about how outcomes were measured/ data were collected.  The lack of transparency in outcome measurement is a concern, but since the readmissions are objective, they are unlikely to be influenced by assessment bias. |
| 1. Bias due to selection of the reported result | Low Risk | No pre-specified statistical plan, but all outcomes were objective and unadjusted, reducing selective reporting risk. |
| Overall risk of bias | Some Concerns | |
| Readmission rate is objective and unlikely to be biased. ITT analysis minimizes bias from deviations and missing data, which is minimal (5 withdrawals in early discharge, 1 in usual care). Although blinding was not possible, this does not significantly impact objective outcomes, and no major evidence of selective reporting was found. | | |

| 2. Skwarska et al., 2000 | | |  |
| --- | --- | --- | --- |
| Risk of Bias (ROB2) | | |  |
| Bias Domain | **Consensus judgement** | **Supporting information** |  |
| 1. Bias due to randomization Process | Some Concerns | The study states that randomization was done using computer-generated numbers. However, it does not clearly state whether the allocation was concealed, which raises concerns. The imbalance in baseline characteristics (e.g., more severe breathlessness in the admitted group) could suggest an issue with randomization, but it could also be due to chance. There is no direct evidence that allocation was compromised. |  |
| 1. Bias due to deviations from intended intervention | Low Risk | It is impossible to blind participants due to the nature of the intervention. However, there is no evidence that deviations occurred in a way that would systematically affect outcomes. The study reports that the results were analysed on an intention-to-treat basis, which further reduces concerns about deviations. |  |
| 1. Bias due to missing outcome data | High Risk | Six patients in the hospital group were lost to follow-up, while none were missing in the home discharge group. The discrepancy raises the possibility of bias, particularly if missingness was related to the true readmission outcome. This could plausibly bias the results, particularly because readmissions may be missed if follow-up was incomplete. |  |
| 1. Bias due to measurement of the outcome | Low Risk | The study does not provide detailed information about how outcomes were measured, making it difficult to determine if there was systematic bias in measurement. However, readmission rate is objective, making measurement bias less likely. |  |
| 1. Bias due to selection of the reported result | Low Risk | Although no pre-specified analysis plan was mentioned, readmission rate is objective. There is no evidence that multiple outcome measures were tested and selectively reported. |  |
| Overall risk of bias | High Risk | |  |
| The study raises concerns about bias, mainly due to unclear allocation concealment in randomization and missing data in the hospital group. While baseline imbalances suggest potential selection bias, this may be due to chance. | | |  |

| 3. Davies et al., 2000 | | |
| --- | --- | --- |
| Risk of Bias (ROB2) | | |
| Bias Domain | **Consensus judgement** | **Supporting information** |
| 1. Bias due to randomization Process | Low Risk | Patients were randomized in a 2:1 ratio using blinded sealed envelopes, and baseline characteristics were comparable. |
| 1. Bias due to deviations from intended intervention | Low Risk | Due to the nature of the intervention, blinding was not feasible. No clear deviations from intervention because of trial context. Some patients were appropriately admitted to hospital following randomisation to hospital at home; this is likely what would have occurred in a real-life setting. |
| 1. Bias due to missing outcome data | Some Concerns | 5% loss to follow-up in the home care group vs. 10% in the hospital group could introduce bias if missing data were related to worsening health or increased risk of readmission. It is possible that patients lost to follow up could have been readmitted and thus missingness depended on its true value, however it is likely that this outcome was obtained through a relatively objective source not prone to this type of bias. Although that is not explicitly mentioned. |
| 1. Bias due to measurement of the outcome | Low Risk | The authors do not provide clear description of how all outcomes were measured.  Readmission rate is objective, and it is unlikely this was ascertained differently between groups.  Limited information provided about how outcomes were assessed and by whom.  Readmission outcome data is unlikely to be biased by non-blinding. |
| 1. Bias due to selection of the reported result | Low Risk | No pre-specified analysis plan provided.  Mostly objective outcomes with a reasonable standardised follow up time and unlikely selected from multiple scales etc.  Basic comparison of readmission numbers presented. Unadjusted analysis. No evidence of multiple subgroup analyses presented. |
| Overall risk of bias | Some Concerns | |
| There were some concerns of bias in this study due to missing data that differed in proportions between arms (10% versus 5%). | | |

| 4. Ojoo et al., 2002 | | |
| --- | --- | --- |
| Risk of Bias (ROB2) | | |
| Bias Domain | **Consensus judgement** | **Supporting information** |
| 1. Bias due to randomization Process | Low Risk | Patients were randomized using sealed envelopes. No significant differences in baseline characteristics were reported. |
| 1. Bias due to deviations from intended intervention | Low Risk | Blinding was not possible due to the nature of the intervention. However, there were no evident deviations from the intended treatment as a result of the trial.  Patients who were readmitted and unable to complete the trial had their results excluded. Since the number of withdrawals was the same in both study arms, this is unlikely to have significantly affected the overall findings. |
| 1. Bias due to missing outcome data | Some Concerns | The study reports that 27 patients in each arm completed the trial, but it is unclear whether any readmission data was missing for them. While there is no strong evidence of bias due to missing data, withdrawals were equal across groups, reducing potential impact. However, missing data might be linked to outcomes, as patients who were readmitted could have had a higher risk of mortality. Despite this, the small sample size and balanced withdrawals make it unlikely that missing data significantly influenced the study’s conclusions. |
| 1. Bias due to measurement of the outcome | Low Risk | There is limited detail about how outcomes were measured.  It is not clear if outcome assessors were aware of the intervention received.  Outcomes were predominantly objective and unlikely to be substantially impacted by knowledge of intervention. |
| 1. Bias due to selection of the reported result | Low Risk | No pre-specified analysis plan is mentioned. However, the study uses objective outcomes, and there is no clear evidence of selective reporting. |
| Overall risk of bias | Some Concerns | |
| There is limited detail provided about how outcomes were assessed and the blinding procedure.  There is some missing data due to withdrawal, although equal in both arms of the trial. It is unlikely that this would significantly change the readmission rates reported by the study. | | |

| 5. Díaz Lobato et al., 2005 | | |
| --- | --- | --- |
| Risk of Bias (ROB2) | | |
| Bias Domain | **Consensus judgement** | **Supporting information** |
| 1. Bias due to randomization Process | Some Concerns | The study states that randomization occurred but does not describe the method. However, baseline characteristics between groups appear similar, reducing risk. |
| 1. Bias due to deviations from intended intervention | Low Risk | Unable to blind due to nature of the intervention.  No clear evidence that there were deviations from the intended intervention due to trial context.  Only one patient in the HH group was readmitted, suggesting minimal cross-over. Also, the study does not indicate major protocol deviations in either group. |
| 1. Bias due to missing outcome data | Some Concerns | The paper does not provide detailed information on missing data. Readmission rate was reported as similar between groups, reducing concern, but missing data handling is unclear. |
| 1. Bias due to measurement of the outcome | Low Risk | Limited information provided about how readmission data was collected. However, readmission rate is objective, and it is unlikely this was ascertained differently between groups. |
| 1. Bias due to selection of the reported result | Low Risk | No pre-specified analysis plan available. |
| Overall risk of bias | Some Concerns | |
| Overall, the study has some concerns mainly due to limited reporting of the randomisation process and unclear handling of missing data. | | |

| 1. Nissen et al., 2007 | | |
| --- | --- | --- |
| Risk of Bias (ROB2 - Cluster) | | |
| Bias Domain | **Consensus judgement** | **Supporting information** |
| 1. Bias due to randomization Process | Low Risk | Randomisation was performed blinded by the project nurse. Baseline characteristics were balanced​. |
| 1. Bias due to deviations from intended intervention | Low Risk | Not possible to blind due to nature of intervention. The number of readmissions during the home treatment period was low (2 patients, 9%)​. No evidence that deviations occurred due to the trial context. |
| 1. Bias due to missing outcome data | Low Risk | Missing outcome data is not explicitly reported, but all randomized patients are included in analysis tables. |
| 1. Bias due to measurement of the outcome | Low Risk | Outcomes were measured at inclusion and follow-up. The assessors likely knew the intervention group, but bias is unlikely as readmission rate is objective outcome. |
| 1. Bias due to selection of the reported result | Low Risk | The study mentions a sample size calculation but does not reference a published protocol or analysis plan. There is no evidence of selective reporting or multiple analyses​. |
| 1. Overall risk of bias | Low Risk | |
| The identified concerns are minor and unlikely to have materially affected the readmission results. | | |

| 1. Ricauda et al., 2008 | | |
| --- | --- | --- |
| Risk of Bias (ROB2) | | |
| Bias Domain | **Consensus judgement** | **Supporting information** |
| 1. Bias due to randomization Process | Low Risk | The study states that randomization was conducted using computer-generated numbers, and allocation was concealed (sealed envelopes). Similar baseline patient characteristics |
| 1. Bias due to deviations from intended intervention | Low Risk | Not possible to blind due to nature of intervention.  No evidence of deviations from intended intervention because of trial context.  Authors state that results analysed on intention to treat basis. |
| 1. Bias due to missing outcome data | Low Risk | The authors report that 3 patients were lost to follow up. However, the numbers lost to follow up in each group were similar so it is unlikely to make a significant difference to observed readmission trends. |
| 1. Bias due to measurement of the outcome | Low Risk | A 6-month follow up evaluation was performed. However, it is unclear how readmission data was collected.  It is unlikely that measurement of the readmission differed in groups.  Outcome assessors were unaware of the intervention group. |
| 1. Bias due to selection of the reported result | Low Risk | The authors state that sample size calculations were used but there is no pre-specified analysis plan provided.  There is no evidence that multiple outcome measurements/ times were used.  There is no evidence that multiple analyses were performed. |
| Overall risk of bias | Low Risk | |
| The identified concerns are minor and unlikely to have materially affected the readmission results. | | |

| 1. Utens et al, 2012 | | |
| --- | --- | --- |
| Risk of Bias (ROB2 - Cluster) | | |
| Bias Domain | **Consensus judgement** | **Supporting information** |
| 1. Bias due to randomization Process | Low Risk | The paper clearly states: Randomisation sequence generated by computer with allocation in sealed envelopes.  Comparable baseline characteristics |
| 1. Bias due to deviations from intended intervention | Low Risk | Blinding not possible due to nature of intervention.  No clear evidence of deviation from intended intervention because of trial context  Authors report that ITT was used. |
| 1. Bias due to missing outcome data | High Risk | There was a significant number of patients that were lost to follow up, The dropout rate was 16% overall, with 25% in the usual care group and 10% in the early discharge group.  Proportion of patients lost to follow up differs in intervention and control groups so may introduce bias in readmission outcomes. |
| 1. Bias due to measurement of the outcome | Low Risk | Outcome measurement was standardized, using the same procedures for both groups. Readmission rate unlikely to be influenced. Low risk. |
| 1. Bias due to selection of the reported result | Low Risk | Pre-specified analysis plan in trial protocol.  Pre-specified outcomes  Pre-specified analysis. |
| Overall risk of bias | High Risk | |
| Overall high degree of bias due to the number of patients that withdrew and were lost to follow up. The proportions were different between the intervention and control group suggesting bias for readmission. | | |

| 1. Jakobsen, et al, 2015 | | |
| --- | --- | --- |
| Risk of Bias (ROB2) | | |
| Bias Domain | **Consensus judgement** | **Supporting information** |
| 1. Bias due to randomization Process | Low Risk | Randomization was external, with sealed opaque envelopes and concealed allocation. |
| 1. Bias due to deviations from intended intervention | Low Risk | Not possible to blind due to nature of intervention.  Intention-to-treat analysis was conducted​. |
| 1. Bias due to missing outcome data | Low Risk | Missing data were substantial for respiratory function, but readmission data were collected for all randomized patients using hospital records and scheduled follow-up visits, and no patients were lost to follow-up within 180 days. Therefore, missing data do not bias the readmission outcome. |
| 1. Bias due to measurement of the outcome | Low Risk | Outcomes were measured following a protocol, and assessors were blinded to group allocation during data analysis​. Readmission is objective and likely accurately captured. |
| 1. Bias due to selection of the reported result | Low Risk | Published trial protocol available  No evidence multiple outcome measurements used  No evidence multiple analyses performed. |
| Overall risk of bias | Low Risk | |
| The main concern is the missing outcome data for respiratory function. However, this issue is specific to one domain and does not impact readmission outcomes. | | |

| 1. Echevarria et al., 2018 | | |
| --- | --- | --- |
| Risk of Bias (ROB2) | | |
| Bias Domain | **Consensus judgement** | **Supporting information** |
| 1. Bias due to randomization Process | Low Risk | External independent randomisation with minimisation, researchers blind to allocation. |
| 1. Bias due to deviations from intended intervention | Low Risk | Not possible to blind due to nature of intervention.  Intention to treat analysis used. |
| 1. Bias due to missing outcome data | Low Risk | There is some missing data for cost outcomes but authors perform sensitivity analysis. There is also a clear statement about how outcome data was obtained for patients who withdrew from the trial for other outcomes. Low risk that missingness affected readmission results. |
| 1. Bias due to measurement of the outcome | Low Risk | Clear description of outcome measurement for all outcomes including readmission and how outcomes were measured if patients withdrew.  Outcome measure same in both groups.  Unclear if outcome assessors were blind to intervention for all outcomes  Readmission rate is objective and unlikely to be biased. |
| 1. Bias due to selection of the reported result | Low Risk | Pre-specified outcomes/ analysis and trial registered.  No evidence of multiple outcome measures.  No evidence of multiple analyses of the data. |
| Overall risk of bias | Low Risk | |
| This study is at low risk of bias across all domains. | | |

1. **Newcastle-Ottawa Scale (NOS) – for Observational Cohort Studies.**

| - Study No. 8: Ansari et al., 2009 |
| --- |

**1. Selection (4 points maximum)**

**1. Representativeness of the exposed cohort:**

- *Selected group of users* (Frequent exacerbators managed by UCT) – **0 stars**

**2. Selection of the non-exposed cohort:**

- Limited description of hospital cohort derivation – **0 stars**

**3. Ascertainment of exposure:**

- Clear documentation of UCT treatment (structured clinical records) – **1 star**

**4. Demonstration that outcome of interest was not present at start of study:**

- Not applicable as both cohorts were experiencing AECOPD at entry – **0 stars**

**Total for Selection: 1 star out of 4**

**2. Comparability (2 points maximum)**

**1. Comparability of cohorts on design or analysis:**

- No mention of controlling for confounders such as disease severity or comorbidities (e.g., via matching or statistical adjustments) – **0 stars**

**Total** **for Comparability: 0 stars out of 2**

**3. Outcome (3 points maximum)**

**1. Assessment of outcome:**

- It is unclear whether outcome assessors were blinded (no mention of independent or blinded assessment) – **0 stars**

**2. Follow-up long enough for outcomes to occur:**

- Follow-up period of 2–3 months is adequate to assess recovery from AECOPD – **1 star**

**3. Adequacy of follow-up:**

- No clear statement on loss to follow-up or handling of missing data – **0 stars**

**Total for Outcome: 1 star out of 3**

| **Category** | **Subcategory** | **Stars Awarded** | **Max Stars** | **Total stars in each category** | **Total Number of stars (out of 9)** |
| --- | --- | --- | --- | --- | --- |
| **Selection** | Representativeness of the Exposed Cohort | 0 | 1 | 1 | 2 |
|  | Selection of the Non-Exposed Cohort | 0 | 1 |  |  |
|  | Ascertainment of Exposure | 1 | 1 |  |  |
|  | Demonstration that Outcome was not Present at Start | 0 | 1 |  |  |
| **Comparability** | Comparability of Cohorts | 0 | 2 | 0 |  |
| **Outcome** | Assessment of Outcome | 0 | 1 | 1 |  |
|  | Was Follow-Up Long Enough for Outcomes to Occur | 1 | 1 |  |  |
|  | Adequacy of Follow-Up of Cohorts | 0 | 1 |  |  |
