# Supplementary material for: What is the evidence for virtual wards or hospital-at-home care pathways for exacerbations of chronic obstructive pulmonary disease? A systematic review and meta-analysis
Source: BMJ Open Respir Res. 2026 Apr 10;13(1):e003611. doi: 10.1136/bmjresp-2025-003611 (PMC13084816; doi:10.1136/bmjresp-2025-003611)
Supplement: online supplemental file 2 [file bmjresp-13-1-s002.pdf]

Supplementary File 2 – Tables and Figures

Study	Description of the intervention	Patient selection criteria	Planned duration of intervention	Escalation criteria for admission	Main clinical outcomes measured	Males, (%)		Age in years (Mean + SD)	
						I	UC	I	UC
**Cotton et al.**	Sent home ideally within 3 days of admission.<br>Visited by a nurse on first morning after discharge and thereafter depending on nurse assessment.<br>Nurse assessed observations and symptoms (no set tool)<br>Medications prescribed by medical team prior to discharge.<br>No other MDT input.<br>Reviewed at 2 months.	Patients being treated for ECOPD<br>No respiratory acidosis<br>No other requirements given	Not given	No specific criteria given.	1. Length of initial admission<br>2. Readmission rate over 60 days<br>3. Number of days in hospital for readmissions.<br>4. Death in 60 days	19 (46%)	16 (40%)	65.7 ± 1.6	68.0 ± 1.2
**Skwarska et al.**	Discharged with treatment package (antibiotics, corticosteroids, nebuliser and oxygen concentrator if needed on loan).<br>Visited by a nurse on first morning after discharge and thereafter every 2-3 days.<br>Changes in prescription by GP.<br>Assessed in own home at 8 weeks.	Patients being treated for ECOPD<br>No confusion, acidosis or new CXR changes	No set criteria – when deemed medically fit.	Not given	1. Readmission in 8 weeks<br>2. Death before discharge and in 8 weeks<br>3. Median days under hospital care<br>4. GP visits per 100 days	63 (51.6%)	24 (38.7%)	68.5 $^{\int}$	69.9 $^{\int}$

**Davies et al.**	Patients escorted home by nursing staff<br>Social support was available if needed.<br>Nebulised therapies, corticosteroids and antibiotics provided.<br>Nurse visited the patient at home morning and evening for 3 days and thereafter at nurse discretion.	Spirometry confirmed COPD<br>Mini Mental Score >7<br>Pulse rate < 100 bpm<br>Systolic BP > 100mmHg<br>pH > 7.35<br>PaO2 > 7.3 kPa<br>PCO2 < 8 kPa<br>Total WCC 4 – 20 x 109/l	Not given	Not given	1. Number of readmissions in 2 weeks<br>2. Number of readmissions in 3 months<br>3. Changes to FEV1 at day 14 and 3 months<br>4. Health related QoL on admission and at 3 months	45 (45%)	30 (60%)	70 ± 8	70 ± 8
**Ojoo et al.**	Patients sent home within 48 hours of admission<br>Discharged with treatment package (antibiotics, corticosteroids, nebuliser and oxygen if needed).<br>Visited daily at home by nursing staff.	Spirometry confirmed COPD<br>Symptoms of ECOPD<br>Lives within 15 miles of hospital	Not given	Not given	1. Satisfaction with care<br>2. Improvement in FEV1<br>3. Improvement in FVC<br>4. Improvement in symptom score<br>5. Readmissions per patient at 3 months	16 (53.3%)	15 (50%)	69.7[∫]	70.1 [∫]

		No respiratory acidosis, type 2 respiratory failure or changes on CXR			6. Readmission rate at 3 months<br>7. Deaths at 3 months				
**Díaz Lobato et al.**	At day 3, discharged to home and visited by Respiratory senior physician and respiratory nurse. Physician visits 3 times during HaH placement. Nurse visits every 12 hours, daily. 24-hour phone access to nurses for consultation.<br>After discharge from HaH, patients were scheduled for a follow-up reassessment at the hospital outpatient clinic 1 month after discharge.	Diagnosis of COPD<br>ECOPD requiring hospitalisation<br>Age < 85 years<br>Lives within 20kM of hospital<br>Telephone<br>Suitable family support<br>At day 3, clinical stability assessed using Borg score,	Not given	Not given	1. Number of therapeutic failures (For UC group: need for admission to ICU, clinical deterioration requiring change in management or nosocomial failure. For I group requirement to come into hospital).<br>2. Calls for medical assistance<br>3. ECOPD at 1 month<br>4. Smoking cessation<br>5. Bed days	17 (85%)	17 (85%)	66 ± 9	66 ± 9

		glycaemic control, Haematocrit, PEF improved from day 1, ECG, CXR and arterial blood gases							
**Nissen et al.**	Patients randomized to home treatment were discharged within 48 hours of admission. They received care at home from a specialized nurse following a protocol that included regular visits to monitor health status, administer treatment, and provide education. The nurse visited the patients every 12 hours to coincide with medication administration. Patients had 24-hour phone access to the nurse for consultations.	ECOPD requiring hospitalisation<br>No pneumonia<br>Not severe exacerbation which requiring intravenous treatment, non-invasive ventilation, or mechanical ventilation.<br>No cardiac or	Not given	Not given	1. Re-admission rate: Measured within two months after discharge (primary outcome).<br>2. Mortality rate: Evaluated during the follow-up period.<br>3. Complication rate: Includes respiratory deterioration, peripheral edema, pneumonia, and cardiovascular instability.<br>4. Length of hospital stay: Compared between home treatment and hospital care groups.<br>5. Resource utilization: Number of home visits, phone contacts, and	12 (55%)	15 (68%)	69 ± 10.3	69 ± 10.1

		psychiatric conditions			healthcare professional time per patient.				
**Ricaud a et al.**	Transferred home after baseline assessments<br>Comprehensive geriatric assessment at home including physiotherapy and occupational therapy.<br>Physician and nurse visit daily for "first days"<br>Then physician visit every 2 – 3 days with daily nurse visits.<br>24-hour help line with urgent visits<br>6 month follow up at home	Patients aged 75 and over<br>ECOPD<br>Requiring hospitalisation<br>No severe hypoxaemia or acidosis.<br>No significant severe co-morbidities and no advanced dementia	Not given	Not given	1. Hospital readmission at 6 months<br>2. Mortality at 6 months<br>3. Depression status, functional status, cognitive status, QoL, nutritional status.<br>4. Carer stress levels<br>5. Patient satisfaction	29 (56%)	39 (75%)	80.1 ± 3.2	79.2 ± 3.1
**Ansari et al.**	Placed on HaH services from referral from community<br>Visited by nurses at home with target of within 30 minutes for initial assessment.<br>Nebuliser, oral steroids and antibiotics provided<br>No clear visiting pattern defined after this.	COPD<br>ECOPD<br>No NIV or LTOT<br>No dementia or stroke	Not given	Not given	1. Mortality at 2 months<br>2. Readmission at 2 months<br>3. MRC dyspnoea scale<br>4. Spirometry<br>5. Handgrip<br>6. Baseline Dyspnoea Index<br>7. Pulse oximetry	31 (51.7%)	16 (53.33%)	72.4 ± 9.0	77.3 ±6.7

	Visited at home at 2-3 months	No upper limb deformity.							
**Utens et al**	At Day 3 switch to oral antibiotics and steroids and meter-dose inhalers.<br>Home visits by nurses on each day for 4 days<br>24-hour telephone access to Respiratory wards	COPD<br>ECOPD<br>Age > 40<br>No requirement for NIV or ICU<br>No confusion<br>Adequate social care	3 days in patient plus 4 days care at home	Not given	1. Clinical COPD Questionnaire primary outcome<br>2. Other outcomes include death or readmission beyond 7 days, death or readmissions in 3 months.<br>3. Quality of life measurements	48 (68.6%)	38 (55.1%)	68.3 ± 10.3	67.8 ± 11.3
**Schou, et al. ** **	First 24 hours of admission, trained to use telemedicine equipment<br>Healthcare professional and technician accompanied patient home.<br>Discharged with treatment package (antibiotics, corticosteroids, nebuliser and oxygen if needed).<br>Patient uploaded physiology parameters during virtual clinic assessment.<br>Daily virtual assessment by physician.	ECOPD admitted to hospital<br>Aged > 45 years<br>Moderate to severe COPD<br>pH >7.35<br>Estimated may require hospital for > 2 days<br>No NIV, severe co-	Not given<br>Discharged when slept > 4 hours, no decline in FEV1 and oxygen saturations > 90% either off oxygen or on usual oxygen.	Not given	1. Cognitive function assessed by visual verbal learning, concept shifting test and Stroop colour word test	10 (45%)	8 (37%)	68 ± 11.7	73 ± 9.6

		morbidities or temperature > 38oC<br>Able to comply with study activities.							
**Jakobsen, et al** **	As Schou et al (same study)				1. Treatment failure (readmission within 30 days)<br>2. Mortality<br>3. Need for manual or mechanical ventilation or NIV<br>4. HRQoL<br>5. User satisfaction	11 (37.9%)	11 (39.3%)	70.86 *	70.71 *
**Echevarria et al.**	One or two daily visits from Respiratory Specialist nurses<br>Emergency contact provided 24 hours a day<br>Physiology measures and arterial blood gas if required<br>Oral and intravenous therapies, oxygen, physiotherapy, psychology, occupational therapy and social care available at home.	Low risk ECOPD (DECAF 0-1)<br>Spirometry confirming COPD<br>Age > 35<br>10 or more smoking pack years	Not given	Not given	1. Total cost of health and social care at 90 days.<br>2. Survival, readmission rate and total bed days over 90 days.<br>3. CAT and HADS<br>4. Patient preference	28 (46.7%)	28 (48.3%)	71 ± 9.6	68.7 ± 10.5

		Long term ventilation<br>pH > 7.35<br>PaCO2 < 6 kPa<br>PaO2 > 6 kPa							

Supplementary Table 1: Summary of the study intervention, patient selection criteria, escalation criteria, additional participant information and reported clinical outcomes. **Legend:** $^{\int}$ = no standard deviation reported. * = mean ages calculated using the midpoints of the provided age ranges. I = Intervention. UC = Usual care. ** = the same study published with different outcomes reported across two publications. CXR = Chest radiograph. pH = Potential of Hydrogen (a measure of acidity or alkalinity). PaCO2 = Partial Pressure of Carbon Dioxide in Arterial Blood. PaO2 = Partial Pressure of Oxygen in Arterial Blood. WCC = White Cell Count. ECG = Electrocardiogram. NIV = Non-Invasive Ventilation. LTOT = Long-Term Oxygen Therapy. HaH = Hospital at home. FEV1= forced expiratory volume in one second. FVC =Forced Vital Capacity. QoL = Quality of Life. ICU = Intensive Care Unit. ECOPD = Exacerbation of Chronic Obstructive Pulmonary Disease. CAT = COPD Assessment Test. HADS = Hospital Anxiety and Depression Scale.

Study	Mortality events/total		Readmission events/total	
	I	UC	I	UC
**Cotton et al.**	1/41	2/40	12/41	12/40
**Skwarska et al.**	4/122	7/62	23/122<br>**27/122**	19/62<br>**21/62*** (2 non-respiratory)**
**Davies et al.**	9/100	4/50	31/100<br>**37/100*** **	16/50<br>**17/50*** **
**Ojoo et al.**	1/30	3/30	9/30	12/30
**Díaz Lobato et al.**	0/20	1/20	1/20	0/20
**Nissen et al.**	1/22	0/22	3/22<br>**4/22*** **	7/22<br>**8/22*** **
**Ricauda et al.**	9/52	12/52	17/52	34/52
**Ansari et al.**	0/30	0/60	1/60	Not given
**Utens et al**	1/70	1/69	17/70	17/69
**Jakobsen, Laursen et al***	3/29	4/28	**<br>8/29<br>10/29<br>13/29	**<br>6/28<br>11/28<br>14/28
**Echevarria et al.**	1/60	1/58	22/60	23/58

Supplementary Table 2: Data on the outcomes (Mortality and readmission to hospital rate) of the eleven included studies in the meta-analysis.

Legend: *The readmission-free survival probabilities were converted into the number of readmission events over the total patients in each group, based on the reported percentages. **Readmission in 1 month, 3 months, and 6 months. ***Rates in bold are the total readmissions (respiratory and non-respiratory reasons). Re-admissions were defined as a return to hospital following discharge from VW/ HaH or hospital. Patients who returned to hospital prior to discharge from VW/ HaH were not included in re-admission figures, where this was reported. Patients who were withdrawn or lost to follow-up have been retained in the denominator as per ITT.

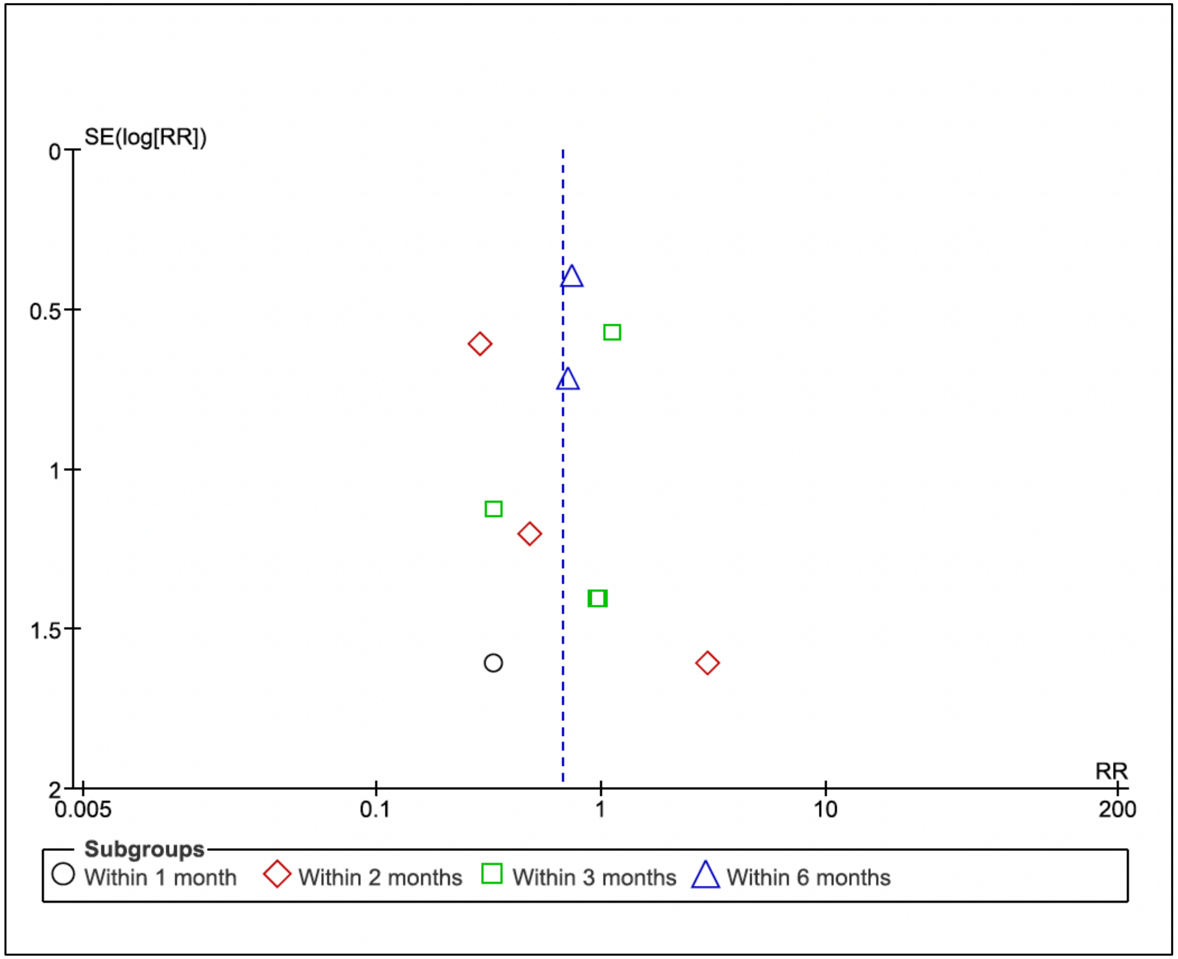

Supplementary Figure 1: Funnel plot of the included studies assessing mortality rate

Legend: within one month (Circles), within two months (Diamonds), within 3 months (squares) and 6 months (triangles). Overall, the distribution appears more symmetrical, indicating a lower probability of publication bias.

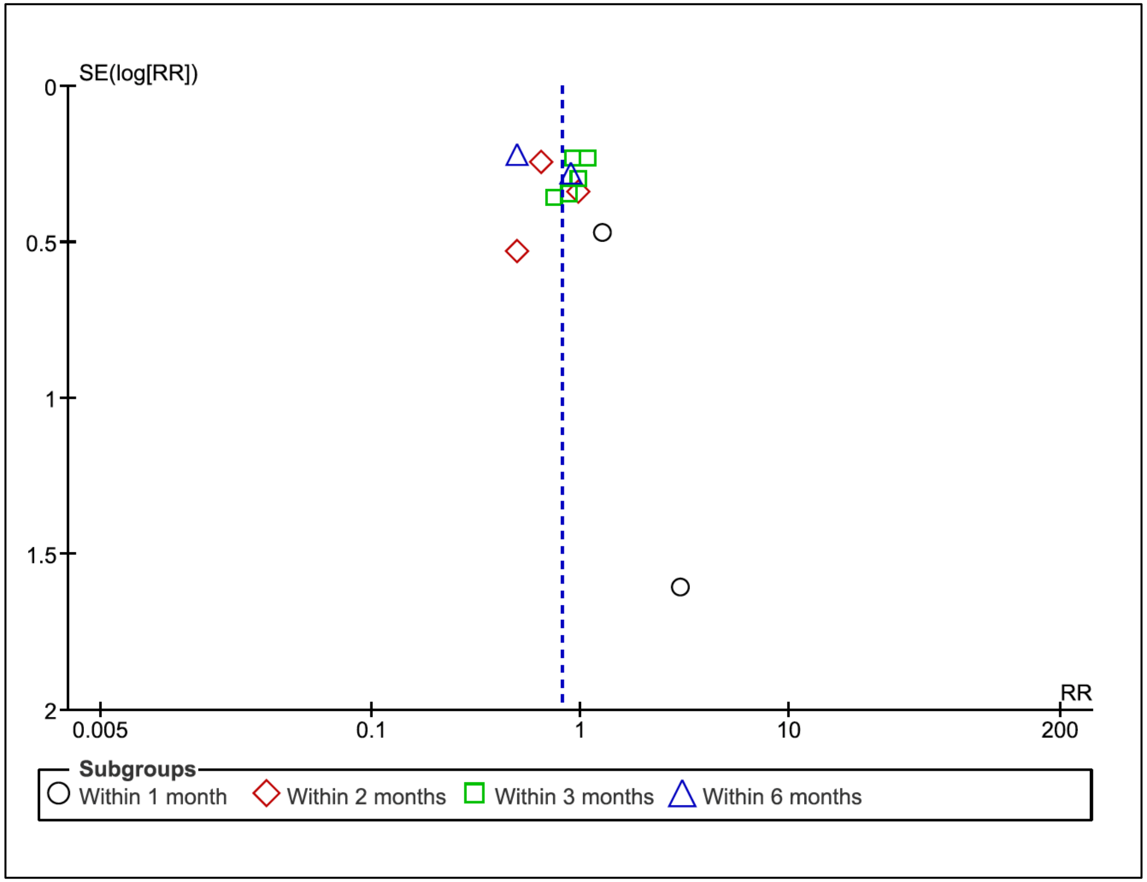

Supplementary Figure 2: Funnel plot of the included studies assessing readmission rate

Legend: within one month (Circles), within two months (Diamonds), within 3 months (squares) and 6 months (triangles). Overall, the plot shows slight asymmetry and some spread, potentially indicating a risk of publication bias.
